# A translation enhancer element from black beetle virus engages yeast eIF4G1 to drive cap-independent translation initiation

**DOI:** 10.1038/s41598-021-82025-6

**Published:** 2021-01-28

**Authors:** Brandon M. Trainor, Arnab Ghosh, Dimitri G. Pestov, Christopher U. T. Hellen, Natalia Shcherbik

**Affiliations:** 1 Department of Cell Biology and Neuroscience, School of Osteopathic Medicine, Rowan University, 2 Medical Center Drive, Stratford, NJ 08084 USA; 2 Graduate School of Biomedical Sciences, Rowan University, 42 E. Laurel Road, Suite 2200, Stratford, NJ 08084 USA; 3 Department of Cell Biology, State University of New York Downstate Health Sciences University, 450 Clarkson Avenue MSC 44, Brooklyn, NY 11203 USA; 4 Present Address: Center for Gene Regulation in Health and Disease, Cleveland State University, 2121 Euclid Ave, Cleveland, OH 44115 USA

**Keywords:** Biochemistry, Genetics, Molecular biology

## Abstract

Cap-independent translation initiation plays crucial roles in fine-tuning gene expression under global translation shutdown conditions. Translation of uncapped or de-capped transcripts can be stimulated by Cap-independent translation enhancer (CITE) elements, but the mechanisms of CITE-mediated translation initiation remain understudied. Here, we characterized a short 5ʹ-UTR RNA sequence from black beetle virus, BBV-seq. Mutational analysis indicates that the entire BBV-seq is required for efficient translation initiation, but this sequence does not operate as an IRES-type module. In yeast cell-free translation extracts, BBV-seq promoted efficient initiation on cap-free mRNA using a scanning mechanism. Moreover, BBV-seq can increase translation efficiency resulting from conventional cap-dependent translation initiation. Using genetic approaches, we found that BBV-seq exploits RNA-binding properties of eIF4G1 to promote initiation. Thus, BBV-seq constitutes a previously uncharacterized short, linear CITE that influences eIF4G1 to initiate 5′ end-dependent, cap-independent translation. These findings bring new insights into CITE-mediated translational control of gene expression.

## Introduction

Translation initiation is one of the most critical regulatory steps in protein synthesis, providing control over individual mRNAs’ translational efficiency and playing an important role in stress responses^1–5^. Untranslated regions at the 5ʹ end of RNA (5ʹ-UTRs), also known as leader sequences, play key roles in translation initiation, inviting intensive investigation to elucidate every aspect of their function.

5ʹ-UTRs are non-coding regions within mRNAs that are located directly upstream of open reading frames (ORF). These elements vary widely in their length and structure^6^. Undoubtedly, the most important function of 5ʹ-UTRs is the recruitment of multiple translation initiation factors required for ribosomal subunit association at the correctly selected AUG start codon. Modifying the 5ʹ-end of mRNA with a 7-methyl guanosine cap structure (m7G-cap) enables it to interact with the cap-binding protein eIF4E, followed by recruitment of the scaffolding factor eIF4G and DEAD-box RNA helicase eIF4A (the eIF4F cap-binding complex)^5,7,8^. eIF4F-primed mRNA associates with the 43S pre-initiation complex (43S PIC) composed of a 40S ribosomal subunit bound to eIF1, eIF3, eIF2, and initiator methionyl-tRNA (tRNA_i_^Met^), forming a scanning-competent 48S initiation complex (48S IC)^4^. 48S IC scans mRNA until it recognizes the first AUG triplet in favorable context, and a codon-anticodon interaction is formed^2^, leading to weakening of ribosomal association of eIF1^9,10^. The action of eIF5B evicts the initiation factors except for eIF1A during subunit joining^11^, and the subsequent hydrolysis of eIF5B·GTP leads to dissociation of eIF5B·GDP and eIF1A and the formation of the elongation-competent 80S complex.

5ʹ-UTRs not only provide the platform for cap-dependent translation initiation, but also possess multiple regulatory functions as modulators of translation initiation, ultimately contributing to the expression of a particular protein at a particular time in a particular environment (reviewed in^6^). For example, the Translation Initiator of Short 5ʹ-UTR (TISU) elements, that are only 12 nucleotides long, promote scanning-free initiation^12^. Other unstructured (linear) elements of 5ʹ-UTRs include upstream ORFs (uORFs) that lead to reduced expression of the downstream primary ORF^13,14^, while the mammalian Kozak sequence (5ʹ-GCC(A/G)CCAUGG-3ʹ) flanking the initiation codon promotes its recognition by the scanning complex to ensure fidelity of translation initiation^4,15,16^. Higher-order RNA structures include internal ribosome entry sites (IRESs, discussed in detail below).

In addition to the conventional cap-dependent mechanism, translation initiation can occur via cap-independent, 5′ UTR-driven processes. The best studied alternative mode of initiation is IRES-dependent translation initiation^17,18^. Most known IRESs derive from viruses that hijack the host’s translational machinery to promote expression of viral proteins^19,20^. They have complex secondary structures^19^ that serve to recruit ribosomal complexes *internally*^21,22^. Depending on the type, IRESs require no or only a few translation factors^23^. IRESs are also present in 5ʹ-UTRs of select cellular transcripts^24,25^. Unlike viral IRESs, cellular IRESs may exhibit weak secondary structures^26^, suggesting that RNA structure is not the sole determinant of the IRES activity. The functional role of cellular IRESs has been linked to cell survival, stress response, differentiation, mitosis, and apoptosis, to provide an alternative way to overcome shutdown of conventional cap-dependent translation initiation^1,27–29^.

Besides IRESs, cap-independent translation initiation can be promoted through cap-independent translation enhancer elements (CITEs). Similarly to IRESs, CITEs can initiate translation without a full set of translation initiation factors^30^. However, unlike many IRES elements, CITEs utilize a scanning mechanism, require the 5ʹ-end of mRNA, and may either carry certain structural features or remain unstructured (reviewed in^31^ and references therein). Engineered mRNA reporters carrying 5ʹ-CITEs taken from viral RNA or mammalian Apaf-1 mRNA have been shown to resist stress-induced translational repression, suggesting that like IRESs, CITEs may provide regulatory functions during unfavorable physiological conditions^30,32,33^. First identified within 3ʹ-UTRs of some plant viruses, CITEs constitute 7 distinct classes, each of which differs greatly in its location (5ʹ-UTR or 3ʹ-UTR), length, and secondary structure, as well as in host factors required for translation initiation (reviewed in^31,34^). A recently published study has found that CITEs exist in eukaryotic cellular mRNAs and participate in fine-tuning expression of stress-response proteins during stress conditions in a cap- and IRES-independent fashion^1^. However, despite their contribution to a plethora of 5ʹ-UTR-regulated initiation mechanisms, CITE elements have received only limited experimental attention.

In this study, we follow up on our previous work and observations by others that showed that a short 39-nucleotide sequence taken from the black beetle virus (BBV; the sequence hereafter termed BBV-seq) can direct efficient translation of a downstream ORF in a wide variety of eukaryotic cells and cell extracts (including insect, yeast and mammalian cells)^35–38^. These data suggested that this viral 5′-UTR exploits a mechanism that is widely used in different eukaryotes. BBV-seq is derived from RNA1 of BBV, a single-stranded, positive-sense RNA virus of the *Nodaviridae* family. For laboratory use, BBV-seq was initially described^39^ as an *“*efficient translation initiation signal” to be used under poor protein expression conditions. As predicted, BBV-seq increased yield in translation of coxsackievirus 3C protease and β-galactosidase^39^. Since the original statement published in 1987 (“The molecular basis of this phenomenon is not well understood”^39^), no published information has appeared concerning the mechanisms of BBV-seq functioning during translation. Given the growing appreciation of the variety of roles that 5ʹ-UTRs play in regulating protein synthesis, we were eager to learn more about the mechanism by which a relatively short BBV-seq influences translation in eukaryotes.

## Results

### BBV-seq promotes reporter protein synthesis in yeast cell-free translation reactions

We began addressing the enigmatic nature of BBV-seq by examining whether this sequence is capable of promoting translation initiation in a cap-independent manner, and whether it functions as an enhancer/regulatory element in conventional cap-dependent translation. To this end, we set up translation reactions using cell-free, translationally competent yeast lysates charged with recombinant RNAs. Given that BBV-seq was previously used as a tool to increase protein expression in yeast cells^38^, we reasoned that the yeast-derived translational system would be an appropriate experimental platform. In addition, the role of individual protein factors in translation could be interrogated by using translation-active extracts prepared from genetically modified yeast strains. Furthermore, we verified that the efficiency of translation driven by BBV-seq in yeast lysates was comparable to that of the widely used Rabbit Reticulocyte Lysate (RRL, see below).

**Figure 1:**
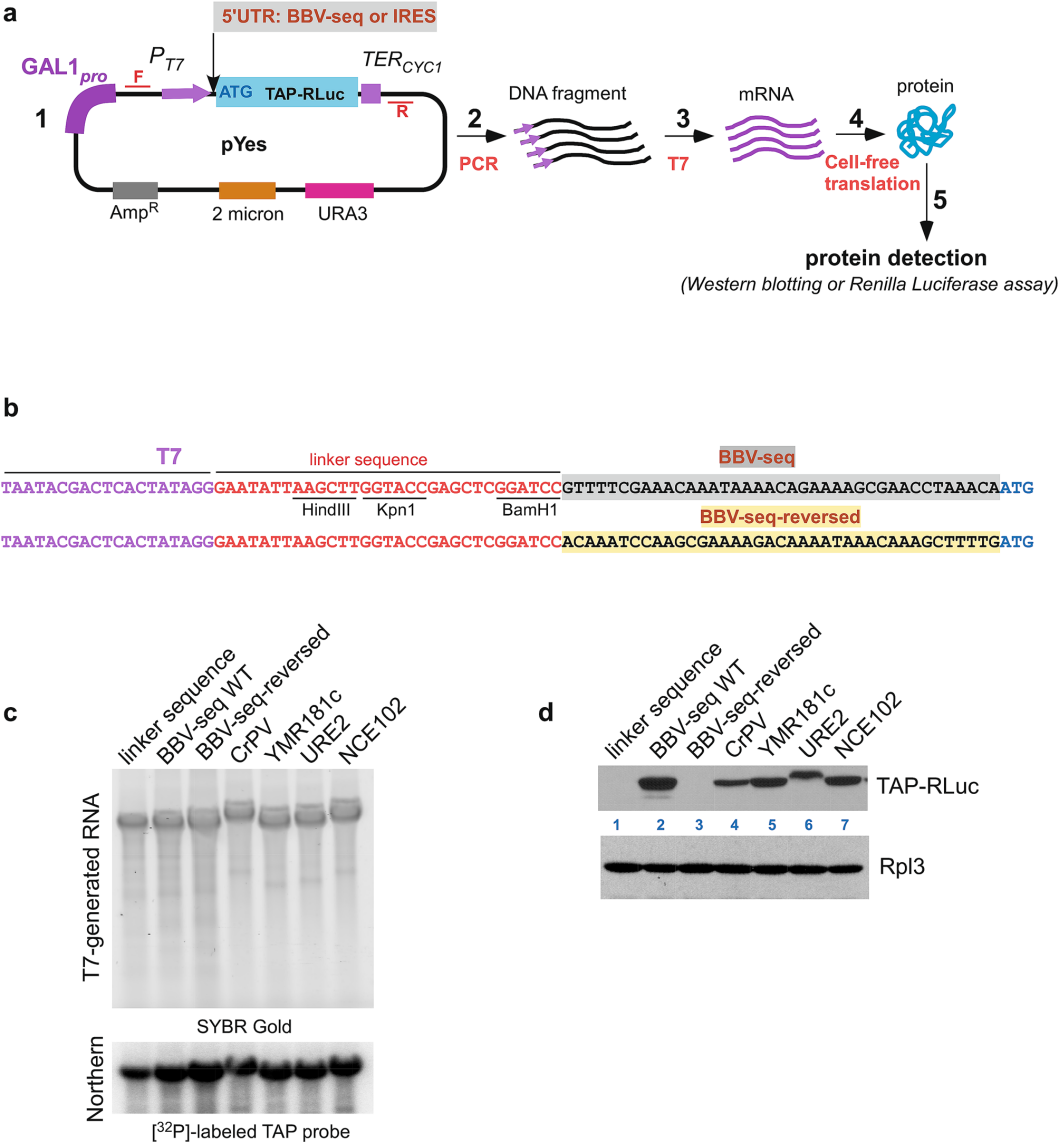
Design and validation of expression of TAP-*Renilla* luciferase (TAP-RLuc) protein reporter under the control of various 5ʹ-UTRs. (**a**) (1) Schematic representation of the pYes-5′-UTR-TAP-RLuc plasmid. A T7 promoter (*P*_*T7*_) and a CYC1 terminator (*TER*_*CYC1*_) flank the TAP-RLuc coding sequence. Insertion of a 5ʹ-UTR upstream of the TAP-RLuc coding sequence is shown. Forward (F) and reverse (R) primers that allow amplification of 5ʹ-UTR-TAP-RLuc are shown in red. The pYes-5ʹ-UTR-TAP-RLuc construct also carries a *URA3* selectable marker, 2µ DNA replication origin, and ampicillin resistance gene (2–5). Workflow of the reporter protein synthesis in the cell-free translation system. Amplified 5ʹ-UTR-TAP-RLuc (2) was used in the T7 polymerase reaction to generate mRNA (3); this mRNA was added to the cell-free translation reaction (4), resulting in protein synthesis and detection (5). (**b**) Sequence of nucleotides located upstream of ATG in the pYes-derived construct from (**a**-1). *From left to right:* T7 promoter sequence (purple) and linker (red) sequences are followed by various BBV-seq sequences (highlighted in gray) or BBV-seq-reverse sequence (highlighted in yellow). (**c**) mRNA generated in (**a**-3) was separated on denaturing 1.2% agarose gel and stained with SYBR Gold (top). RNA was transferred onto nylon membranes and subjected to northern hybridization using a [^32^P]-labeled TAP-specific probe (bottom). (**d**) Translationally competent yeast lysates were charged with mRNA from (**c**) and the reaction products were analyzed by western blotting using anti-TAP antibodies (top) and anti-Rpl3 control antibodies (bottom).

**Figure 2:**
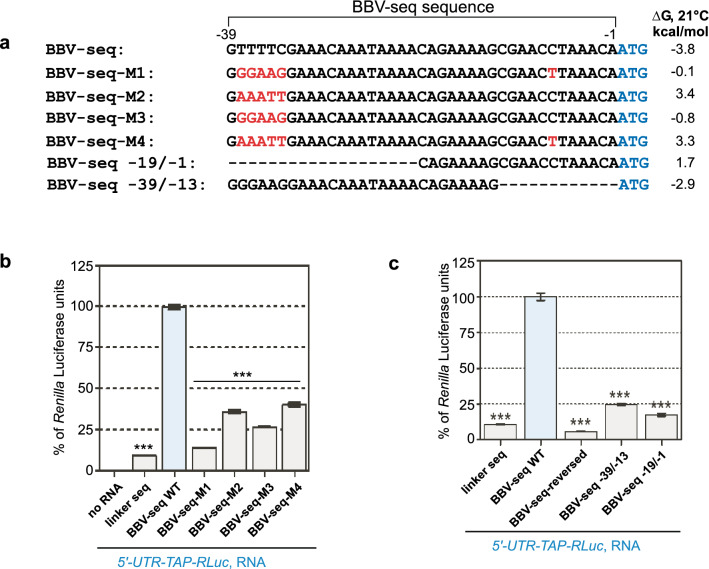
Nucleotide substitutions within BBV-seq result in inhibition of translation in cell-free reactions. (**a**) Sequence alignments of wild-type and mutant BBV-seq sequences relative to the ATG start codon for the respective constructs are shown here for comparison. Gibbs energies (∆G) at 21 °C calculated for each BBV-seq variant are shown on the right. (**b**,**c**) RNAs containing indicated 5′-UTR upstream of TAP-RLuc reporter were used in cell-free translation reactions as described in Fig. 1d. Production of the protein reporter was analyzed using a *Renilla* luciferase assay and is presented as bar graphs. Data represented as % of *Renilla* luciferase units derived from the reaction containing *BBV-seq-WT-TAP-RLuc* mRNA. Error bars represent standard error of the mean (SEM) of three individual experiments. “***” denotes *P*-value < 0.001.

**Figure 3:**
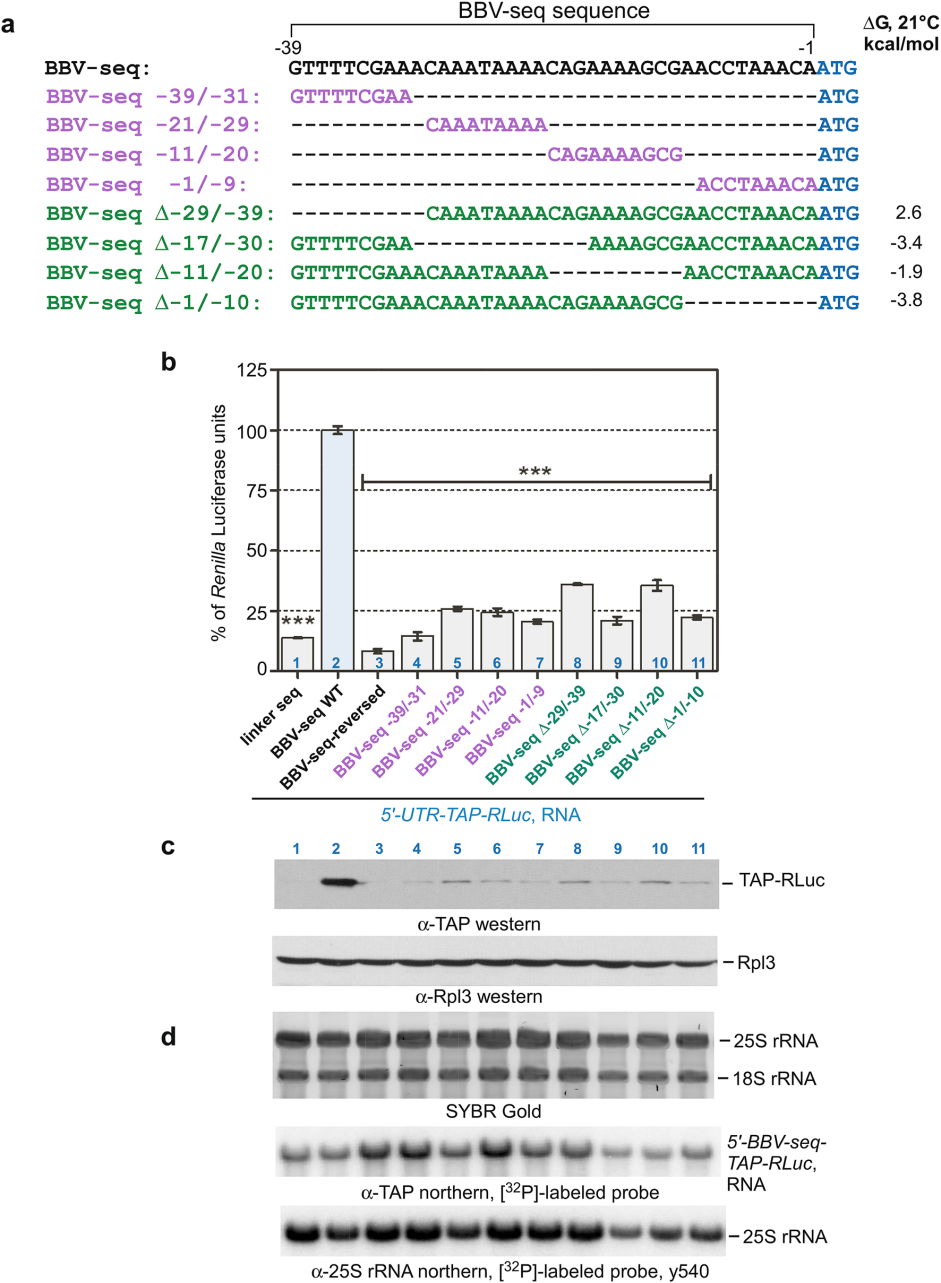
BBV-seq mutational analysis reveals that the entire sequence is required for efficient protein translation in cell-free reactions. (**a**) Sequence alignments of BBV-seq wild-type and deletion mutants. Gibbs energies (∆G) at 21 °C are shown on the right. (**b**–**d**). RNAs containing indicated 5′-UTR upstream of TAP-RLuc reporter were used in cell-free translation reactions as described in Fig. 1d. (**b**) Reaction products were analyzed by a *Renilla* luciferase assay and data are shown as % of *Renilla* luciferase units derived from the reaction containing *BBV-seq-WT-TAP-RLuc* mRNA. Error bars represent standard error of the mean (SEM) of three individual experiments; “***” denotes *P*-value < 0.001. (**c**,**d**) Proteins and RNA were extracted from the luciferase reactions and further characterized by (**c**) western blots using anti-TAP or anti-Rpl3 antibodies and (**d**) northern hybridizations using *TAP*- and 25S rRNA-specific probes. SYBR Gold staining of RNA prior to transfer is shown in (**d**).

**Figure 4:**
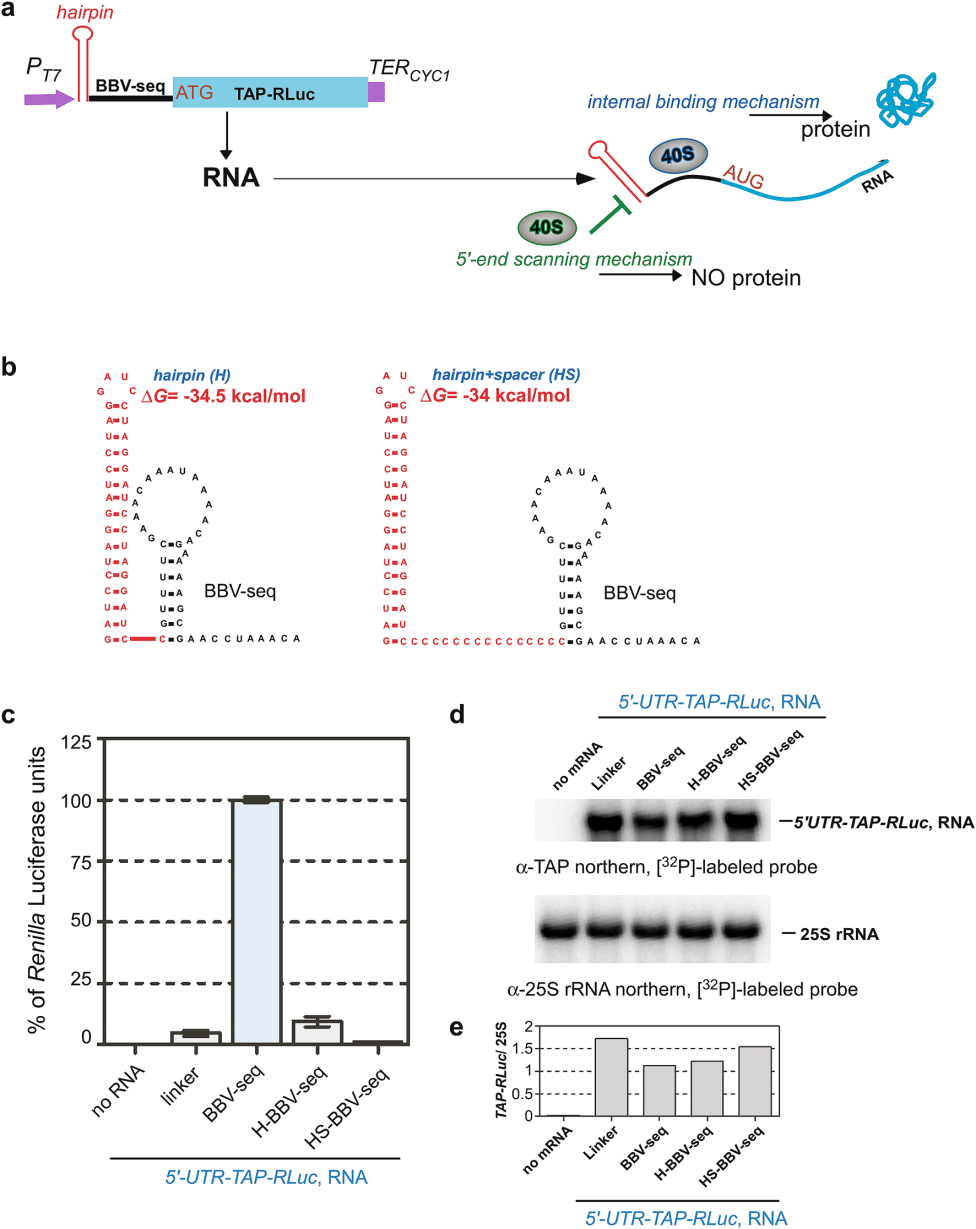
BBV-seq-driven translation initiation occurs via a scanning mechanism in cell-free translation reactions. (**a**) Schematic representation of the strategy used to distinguish between two alternative translation initiation mechanisms: 5ʹ-end scanning vs. internal binding of the 40S subunit (as a part of 43S PIC). Left panel depicts insertion of an RNA loop (marked in red) upstream of the BBV-seq sequence in the pYes-based construct described in Fig. 1a-1. The right panel illustrates that protein synthesis would be blocked by the hairpin at the 5′ end of mRNA when only the scanning mechanism can be used, and that protein synthesis would proceed if translation is initiated internally regardless of the presence of hairpin. (**b**) Secondary structures of the hairpin fused to BBV-seq directly (left) or via an unstructured poly-C spacer (right). (**c**) mRNAs as indicated were translated in vitro using yeast lysates as described in Fig. 1d. Production of the TAP-RLuc reporter was analyzed using a *Renilla* luciferase assay. Data are represented as % *Renilla* Luciferase units derived from the reaction containing *BBV-seq-WT-TAP-RLuc* mRNA. Error bars represent standard error of the mean (SEM) of three individual experiments. (**d**) RNA was extracted from the luciferase reactions and analyzed by northern hybridizations with the indicated probes. (**e**) The *TAP-RLuc*/25S rRNA ratios of hybridization signals in each lane of the blot shown in (**d**).

For RNA synthesis, we first constructed a set of plasmids in which a nucleotide sequence encoding the tandem affinity purification (TAP) protein tag was fused with the gene for *Renilla* luciferase (hereafter, TAP-RLuc); this dual-protein reporter was cloned into the pYes vector plasmid downstream of the T7 promoter, while various 5ʹ-UTRs were placed immediately upstream of the reporter’s ATG start codon (schematic in Fig. 1a-1). The TAP-RLuc reporter allowed us to assess protein production in the cell-free translation reaction by western blotting as well as by *Renilla* luciferase assays (Fig. 1a-4). The BBV-seq sequence was obtained from the pBD7 expression vector^39^. In addition to BBV-seq-TAP-RLuc, we engineered two control constructs which either completely lacked BBV-seq and only had a 31-nt linker at the 5′ end, or contained the BBV-seq in reverse (Fig. 1b). We also generated pYes-based constructs in which TAP-RLuc was under the control of 4 different IRES elements, including CrPV-IRES from cricket paralysis virus^40^ which functions in yeast^41^ and native yeast IRESs URE2-IRES^42^, NCE102-IRES, and YMR181c-IRES^27^ (Fig. 1a-1). Next, we amplified DNA fragments using primers that anneal upstream and downstream of the region of interest (i.e., *P*_*T7*_-5ʹ-UTR-TAP-RLuc, Fig. 1a-2) and used them in the RNA synthesis reaction catalyzed by T7 polymerase (Fig. 1a-3). The quality of generated RNAs was assessed by SYBR Gold staining (Fig. 1c, top) and northern hybridization using a probe specific to the TAP sequence (Fig. 1c, bottom).

Having validated the mRNAs, we next examined protein production using cell-free yeast extracts prepared from wild-type strain BY4741. We added equal amounts of uncapped mRNA transcripts to the yeast lysates, along with the energy regeneration system^43^. Reactions were incubated at 21 °C, consistent with previous studies^42,44,45^, and reaction products were analyzed by western blotting with anti-TAP antibodies. As a loading control, we used antibodies that recognize the Rpl3 protein from the large ribosomal 60S subunit. Strikingly, we found that BBV-seq, but not the linker or reverse BBV sequences, drove expression of TAP-RLuc to levels comparable with IRES-controlled protein synthesis (Fig. 1d). Of note, the URE2-IRES-driven translation resulted in a TAP-RLuc protein product of a slightly higher molecular weight (Fig. 1d, line 6), as URE2-IRES contains an internal AUG located 51 nt upstream of the *TAP-RLuc* AUG, leading to an addition of 17 extra amino acids to the synthesized protein^42^. To further validate the linker-only negative control, we replaced the pyrimidine base (C) at position − 1 relative to the start codon AUG with a purine (A) (see Supplementary Fig. S1), as the − 1 position of BBV-seq is occupied by an A, potentially placing the AUG of the reporter gene in a favorable Kozak context. Using the *Renilla* luciferase assay as a quantitative readout of the reporter gene expression, we found that this C to A replacement did not significantly affect reporter yield (see Supplementary Fig. S1), arguing against the possibility that the − 1 purine creates a favorable Kozak context^46^ under the tested conditions.

Taken together, these data describe the development and validation of an experimental platform to study the effects of the 5′-UTR on mRNA translation in a cell-free yeast system and demonstrate that BBV-seq promotes translation initiation independently of capping by a yet to be discovered mechanism.

### BBV-seq-dependent translation initiation does not rely on structure and requires the full-length BBV-seq nucleotide sequence

As mentioned in the Introduction, a strong secondary structure is not always a prerequisite for IRES-mediated translation initiation^26^. Nevertheless, we next examined whether BBV-seq RNA forms a defined and structurally stable IRES-like module. We were skeptical about this possibility because BBV-seq is very short (39 nucleotides; Figs. 1b, 2a) and likely cannot form structured modules like many viral IRESs^4,47^. For comparison, the CrPV-IRES sequence is ~ 190 nucleotides long and folds into 3 pseudoknots^48^; the 3′-terminal pseudoknot, PK-I, mimics the tRNA/mRNA structure to promote interaction of viral mRNA with a ribosome^47^.

By using the RNA structure tools version 6.0.1, we determined that the most plausible structure in BBV-seq is a large stem-loop at the 5ʹ end of the RNA (see Supplementary Figs. S1, S2). However, given the presence of mostly weak A-U base pairs in the stem (see Supplementary Figs. S1, S2), with a correspondingly low change in Gibbs free energy upon duplex formation (Δ*G* =  − 3.8 kcal/mol, Fig. 2a, Supplementary Table S1), it was evident that BBV-seq does not form stable structures under our reaction conditions. Notably, the linker sequences were predicted to form relatively stable stem-loops with ∆G =  − 8.9 kcal/mol for the (− 1) pyrimidine linker and ∆G =  − 9 kcal/mol for the (− 1) purine linker (see Supplementary Fig. S1, Supplementary Table S1). To examine how these linker structures might affect translation initiation, a different linker sequence lacking stable structure (as predicted by the same algorithm, see Supplementary Fig. S1) was placed 5′ to *TAP-RLuc*. As a positive control, we placed BBV-seq upstream of *TAP-RLuc* in the absence of any additional sequence (see Supplementary Fig. S1). RNAs synthesized from the new constructs were added to translation reactions, and production of TAP-RLuc was examined in comparison with the (− 1) pyrimidine linker-containing *TAP-RLuc* RNA. Using a *Renilla* luciferase assay, we observed no significant difference in the TAP-RLuc translation yields between the (− 1) pyrimidine and unstructured linkers (see Supplementary Fig. S1). Given the lack of a significant effect of the stem-loop of the (− 1) pyrimidine linker on translation initiation, we used it as a negative 5′ UTR control in subsequent experiments.

We next considered that the stimulation of translation by BBV-seq (Fig. 1d) depends on the primary sequence of nucleotides rather than a secondary structure. To test this hypothesis, we constructed a panel of mutants with nucleotide changes and deletions in BBV-seq (Fig. 2a). First, a replacement of T^−38^TTTC^−34^ with G^−38^GAAG^−34^ and C^−7^ with T^−7^ (BBV-seq-M1) or T^−38^TTTC^−34^ with G^−38^GAAG^−34^ (BBV-seq-M3, Fig. 2a) enlarged the size of the 5′-terminal hairpin (see Supplementary Fig. S2). Substituting nucleotides T^−38^TTTC^−34^ with A^−38^AATT^−34^ (BBV-seq-M2, Fig. 2a) reduced the size of the 5ʹ-end hairpin loop (see Supplementary Fig. S2), while a replacement of T^−38^TTTC^−34^ with A^−38^AATT^−34^ and C^−7^ with T^−7^ (BBV-seq-M4, Fig. 2a) resulted in the formation of two small hairpins at the 5′ and 3′ ends of BBV-seq. The stability of the resulting potential structures in these mutants was even lower than in wild-type BBV-seq (Δ*G* =  − 0.1 kcal/mol for M1; 3.4 kcal/mol for M2; − 0.8 kcal/mol for M3, and 3.3 kcal/mol for M4, Fig. 2a, Supplementary Table S1). All mutants were then tested in the cell-free translation reactions, and protein synthesis was assessed using *Renilla* luciferase assays. We detected a significant (over threefold) drop in *Renilla* luciferase activity when M1–4 mutants were used instead of wild-type BBV-seq, indicating a lower capacity to promote protein synthesis (Fig. 2b). To further address whether sequence features affect BBV-seq activity, we constructed two additional mutants, with a truncated 5ʹ-end (BBV-seq − 19/− 1, Fig. 2a) or 3ʹ-end (BBV-seq − 39/− 19, Fig. 2a). RNA structure prediction indicated that both truncation mutants can only form weak stem-loops (see Supplementary Fig. S2), while the luciferase assay showed a significant decrease (over fourfold) in protein production in the cell-free reactions (Fig. 2c). Interestingly, the structure of BBV-seq − 39/− 19 almost completely resembled wild-type BBV-seq, further confirming that this weak stem-loop does not contribute to translation initiation. Consistently, the Δ*G* values determined for the newly generated BBV-seq truncation mutants (1.7 kcal/mol for BBV-seq − 19/− 1 and − 2.9 kcal/mol for BBV-seq − 39/− 19) indicated a lack of stable secondary RNA structures (Fig. 2a, Supplementary Table S1).

To further clarify the region(s) within BBV-seq that are essential for translation initiation, we next examined BBV-seq mutants containing deletions of different lengths (Fig. 3a) using cell-free translation reactions. *Renilla* luciferase assays demonstrated that all these mutants displayed a significantly lower TAP-RLuc production (Fig. 3b). Hypothetically, some (or all) truncated BBV-seq variants may retain the ability to bind initiation factors and thus to recruit 43S PICs, but due to the proximity of AUG in ORF1 (in our case, *TAP*), the 40S subunit might skip this AUG and initiate translation from an AUG located downstream in the coding sequence. In that case, a shorter protein(s) would be generated. To address this possibility, we extracted proteins and RNA from the same samples that were analyzed with the luciferase assays. As expected, western blotting showed a strong TAP-RLuc protein signal in samples derived from the reaction programmed with RNA containing full-length BBV-seq (Fig. 3c, lane 2). Protein products generated from truncated BBV-seq 5′ UTRs (Fig. 3a, colored sequences) were of the same size as with the full-length BBV-seq (Fig. 3c, lanes 4–11), although they were expressed at low levels, consistent with the luciferase assay data (compare Fig. 3b,c). To confirm that the low expression of TAP-RLuc is not due to degradation of the truncated BBV-seq RNAs, we hybridized RNA extracted from the translation reactions with a TAP-specific probe and normalized *TAP-RLuc* RNA-derived signals by a 25S rRNA probe signal (Fig. 3d). The *TAP-RLuc*/25S rRNA ratio (see Supplementary Fig. S3) indicated that all RNAs tested in this experiment maintained similar levels of stability in the translation reaction.

Taken together, these data suggest that all 39 nucleotides of BBV-seq are required for efficient translation initiation of the protein reporter in the translationally-competent yeast cell-free system (Fig. 3).

### Initiation of translation mediated by BBV-seq utilizes a scanning mechanism

Next, we examined whether BBV-seq utilizes a 5ʹ-end scanning mechanism or recruits the 43S PIC internally to initiate translation. Although we did not detect any strong secondary structures in BBV-seq typical for most viral IRESs (see Supplementary Fig. S2, Supplementary Tables S1, S2), the latter possibility could not be ruled out, as some IRESs do not depend on highly-structured elements and are characterized by the presence of specific single-stranded regions^26,27,49^.

We reasoned that in the scanning scenario, blockage of the 5ʹ-end of the BBV-seq mRNA would abolish translation in the cell-free reaction. Conversely, manipulating the 5ʹ-end of mRNA should have no significant effect on the translational reporter yield in the case of the internal recognition of BBV-seq (schematic in Fig. 4a). We inserted a stable hairpin structure with a large negative value of ∆G (Fig. 4b, ∆G =  − 34 kcal/mol)^42,50^ at the extreme 5′-terminus of the *BBV-seq-TAP-RLuc* sequence (Fig. 4b, left), thus blocking 5′-end-dependent binding of the 43S PIC (Fig. 4a). To rule out the possibility that the hairpin’s close proximity to the BBV-seq might obstruct any hypothetical IRES-like internal initiation, we inserted an unstructured spacer between the hairpin and BBV-seq (Fig. 4b, right). No protein reporter synthesis was detected with the hairpin-BBV-seq (H-BBV-seq) or hairpin-spacer-BBV-seq (HS-BBV-seq) constructs, while the BBV-seq RNA lacking the hairpin promoted efficient translation, as determined by the luciferase assay (Fig. 4c). The observed difference in the reporter protein synthesis was not due to hairpin-RNA degradation during the translation reaction, as northern hybridization detected intact *TAP-RLuc* RNAs in all samples tested (Fig. 4d), and the *TAP-RLuc*/25S rRNA ratio, determined as described above, confirmed the stability of the RNAs (Fig. 4e). Another theoretical possibility that the 40S ribosomal subunits are recruited internally by BBV-seq but become stalled due to hairpin seems unlikely because the hairpin constructs containing an unstructured spacer (Fig. 4b, right) showed no TAP-RLuc expression (Fig. 4c). These data suggest that BBV-seq-mediated translation initiation requires the free 5′-end of mRNA for the 43S PIC to start scanning for the start codon.

Given that some well-characterized viral IRESs are not functional in yeast cells or yeast-based cell-free translation extracts, we also considered the hypothetical scenario in which BBV-seq is unable to act as an IRES in our yeast-based translation platform because it lacks necessary *trans*-acting factors. To address this possibility, we assayed the expression of TAP-RLuc in RRL. To restrict initiation on BBV-seq containing mRNAs to the hypothetical internal binding mechanism (Fig. 4a), we used the hairpin-containing *BBV-seq* RNAs (H-BBV-seq and HS-BBV-seq, Fig. 4b) and the hairpin-less control *BBV-seq* RNA. Similar to yeast lysate-based expression (Fig. 4c), BBV-seq promoted efficient TAP-RLuc translation in RRL only when the 5′-end of RNA was not sequestered in a hairpin, while northern hybridization confirmed equal amounts of *TAP-RLuc* RNAs post-reaction (see Supplementary Fig. S4). BBV-seq therefore does not support initiation by internal ribosomal entry in yeast or mammalian cell-free translation extracts in which it is active as a translational enhancer.

**Figure 5:**
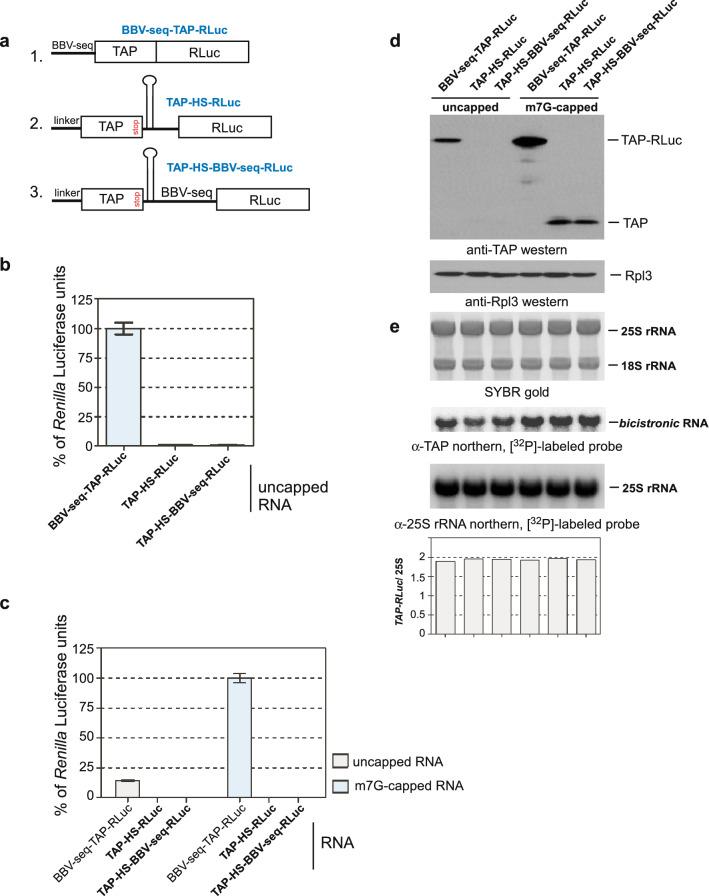
BBV-seq fails to initiate translation when placed upstream of ORF2 (*RLuc*) in a bicistronic construct. (**a**) Schematic representation of the constructs. In bicistronic constructs, ORF1 (*TAP*) and ORF2 (*RLuc*) are separated by the hairpin-spacer-BBV-seq (HS-BBV-seq, construct 3) or hairpin-spacer (HS, construct 2). In monocistronic construct 1, *TAP* is fused with *RLuc* and placed under the BBV-seq 5′-UTR. (**b**,**c**) RNA generated from constructs shown in (**a**) was in vitro m7G-capped or remained unmodified and then translated in cell-free reactions. Reaction products were analyzed by a *Renilla* luciferase assay. Data are presented as % of *Renilla* luciferase units in reactions with uncapped *BBV-seq-TAP-RLuc* mRNA (**b**) or m7G-capped *BBV-seq-TAP-RLuc* mRNA (**c**). (**d**,**e**) Proteins and RNA extracted from the luciferase reactions shown in (**b**,**c**) were analyzed by western blotting with anti-TAP and anti-Rpl3 antibodies (**d**) and northern hybridizations with the indicated probes (**e**). SYBR Gold staining of RNA prior to transfer is shown in (**e**). *TAP-RLuc*/25S rRNA ratios of hybridization signals in each lane are shown.

To provide additional evidence that BBV-seq does not promote translation initiation by acting as an IRES, we constructed a bicistronic reporter, in which BBV-seq was inserted between ORF1 (*TAP*) and ORF2 (*RLuc*). To prevent post-termination ribosomes from reinitiating, we inserted a stable hairpin between the two ORFs upstream of BBV-seq. To rule out the possibility that putative internal entry on this short BBV sequence could be compromised sterically, we placed a spacer between the hairpin and the BBV sequence (schematic in Fig. 5a, construct 3). We used two controls: a bicistronic reporter without BBV-seq between *TAP* and *RLuc* (schematic in Fig. 5a, construct 2), and the BBV-seq-TAP-RLuc fusion used earlier (Figs. 1, 2, 3, 4), in which TAP lacks a stop codon (schematic in Fig. 5a, construct 1). RNAs generated from these constructs (Fig. 5a) were either m7G-capped or unmodified and added to yeast cell-free translation reactions. As before, the synthesis of RLuc was assessed by *Renilla* luciferase assays (Fig. 5b,c), while production of TAP or TAP-RLuc was examined by western blotting using anti-TAP antibodies (Fig. 5d). Northern hybridizations of RNAs extracted from the reactions confirmed the stability of all transcripts used in this experiment (Fig. 5e). Consistent with our previous data (Figs. 1, 2, 3, 4), we detected BBV-seq-mediated expression of the TAP-RLuc fusion from uncapped RNA; however, only background levels of TAP and RLuc were detected with the uncapped bicistronic RNAs (Fig. 5b,d, lanes 1–3). Capping, as expected, significantly increased production of the TAP-RLuc fusion (Fig. 5c, bar 4) but not RLuc derived from either of the bicistronic constructs (Fig. 5c, bars 5–6; Fig. 5d, lines 5–6), suggesting that BBV-seq placed between the two ORFs does not promote initiation of translation. Taken together, these results indicate that BBV-seq lacks an intrinsic IRES activity and likely instead utilizes a scanning mechanism to initiate translation.

### BBV-seq increases translation from capped mRNA

Capping the 5ʹ-ends of newly synthesized transcripts with m7GpppN (m7G) protects mRNAs from rapid degradation in cells and enables recruitment of eIF4E in canonical cap-dependent translation initiation^4^. To compare the stimulatory effects of BBV-seq on translation to those conferred by 5′-end capping, we modified T7 polymerase-synthesized *BBV-seq-TAP-RLuc* and the control *linker-TAP-RLuc* RNAs with the [m^7^G(5′)ppp(5′)G] cap analog (m7G) or kept them unmodified. Equal amounts of these RNAs were added to cell-free translation reactions, and the amounts of translation products were analyzed by quantitative western blotting. For this analysis, we used anti-TAP antibodies to detect full-length TAP-RLuc, with antibodies against ribosomal protein Rpl3 providing normalization of the values obtained in each lane. This double-antibody approach coupled with quantitative near-infrared secondary antibody detection allowed measurements of TAP-RLuc and Rpl3 amounts in the same reactions, thus minimizing experimental variability.

**Figure 6:**
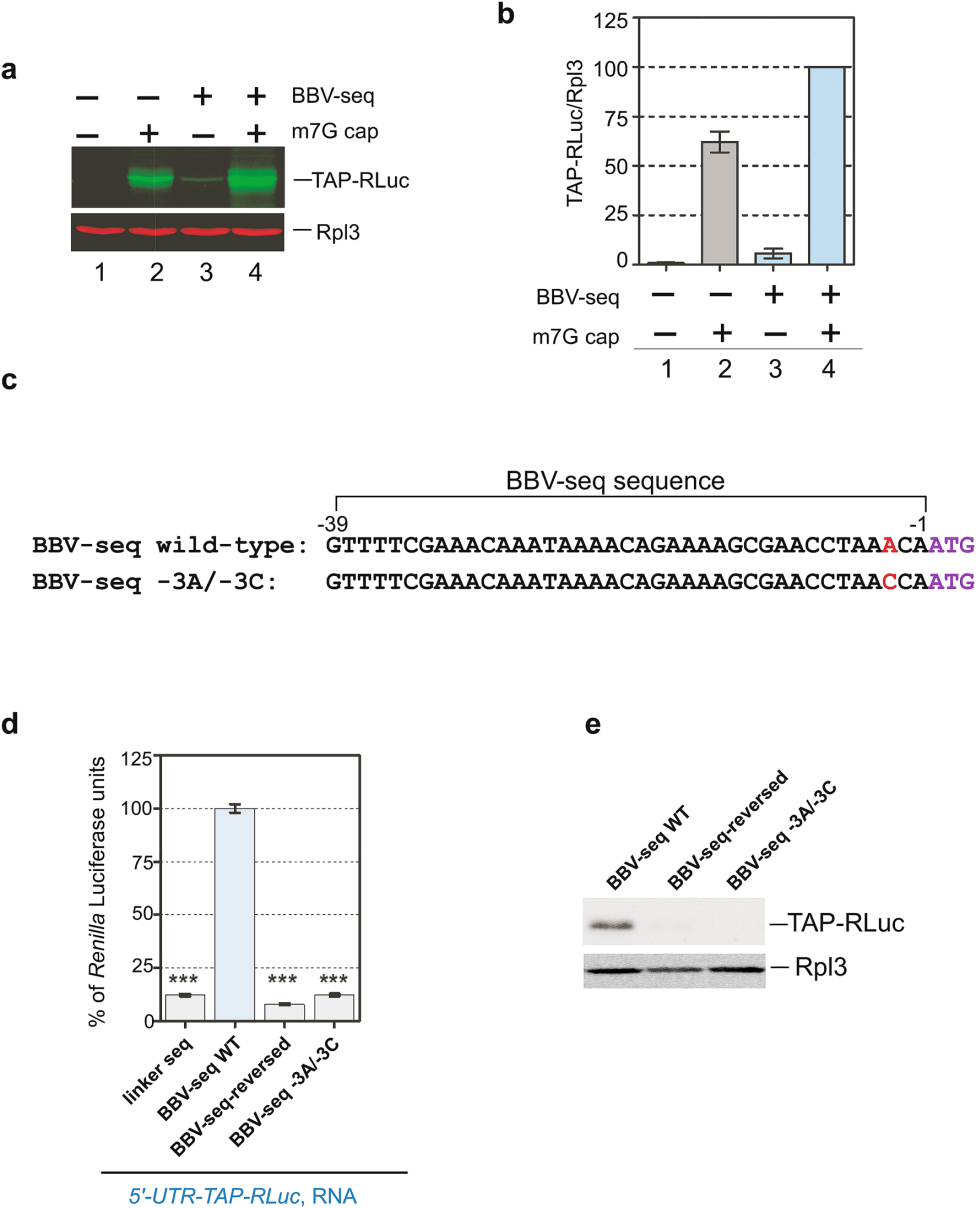
(**a**,**b**) BBV-seq contributes to the efficiency of cap-dependent translation. (**a**) mRNAs with or without BBV-seq were generated as described in Fig. 1a-3 and were capped in vitro (m7G, +) or remained uncapped (−). Capped and uncapped RNAs were translated in cell-free translation reactions, and the translation products were analyzed by quantitative western blotting as described in “Methods” section. (**b**) The fluorescent signal in the TAP-RLuc and Rpl3 bands was used to calculate TAP-RLuc normalized to Rpl3 present in the same reaction. TAP-RLuc/Rpl3 ratios are presented in the graph. (**c**–**e**) Presence of − 3A within BBV-seq 5′-UTR relative to ATG start codon is essential for translation of uncapped mRNA. (**c**) Alignment of wild-type and mutant BBV-seq sequences. In mutant BBV-seq, the single A to C nucleotide substitution was placed in position − 3 (− 3A/− 3C) relative to the ATG start codon in which A is designated as + 1. (**d**) mRNAs indicated in the figure were used in cell-free translation reactions as described in Fig. 1d; protein products were analyzed using a luciferase assay. Data are represented as % *Renilla* Luciferase units derived from the reaction containing *BBV-seq-WT-TAP-RLuc* mRNA. Error bars represent standard error of the mean (SEM) of three individual experiments; “***” denotes *P*-value < 0.001. (**e**) Reaction products from (**d**) were analyzed by western blotting using anti-TAP and control anti-Rpl3 primary antibodies and HRP-fused secondary antibodies. Protein signals were detected using the ECL system.

As expected, no protein reporter translation was detected in reactions with uncapped RNA lacking BBV-seq (Fig. 6a, lane 1), while modifying the 5ʹ-end of mRNA with the m7G cap analog strongly increased TAP-RLuc synthesis (Fig. 6a, lane 2). The presence of BBV-seq in the 5ʹ-UTR was sufficient for translation to occur with uncapped RNA (Fig. 6a, lane 3). Interestingly, the combination of capping and BBV-seq resulted in the strongest TAP-RLuc signal (Fig. 6a, lane 4). Quantification of the reporter levels showed that the presence of BBV-seq enhanced protein synthesis from capped transcripts ~ 1.7-fold (Fig. 6b).

Taken together, these data indicate that BBV-seq can improve translation efficiency in the context of cap-dependent initiation, suggesting that BBV-seq and the 5′-cap have additive stimulatory effects on translation initiation.

### An adenine at position − 3 is essential for BBV-seq-driven translation initiation

One way to explain the BBV-seq-mediated 1.7-fold increase in reporter levels detected with in vitro-capped RNA (Fig. 6a,b) is that BBV-seq might serve as a translational enhancer during cap-mediated translation initiation. In vertebrates, placing the first AUG triplet in the optimum context of the Kozak sequence (5ʹ-GCC(A/G)CC**AUG**G-3ʹ) improves correct start codon selection. The mammalian Kozak sequence contains a purine in − 3 and G in + 4 positions (the A of the AUG codon is designated + 1)^16,51^. In *S. cerevisiae*, recent studies identified a strong requirement for an A present in the − 3 position relative to the start AUG codon^52–54^. To address the possibility of BBV-seq providing the optimum start codon context in cap-dependent translation, we generated a BBV-seq mutant that carries a − 3A to − 3C nucleotide substitution relative to the start codon (Fig. 6c). Strikingly, this resulted in a ~ tenfold reduction in TAP-Luc translation as determined by luciferase assays (Fig. 6d) and western blotting (Fig. 6e). Thus, the − 3A is necessary for BBV-seq to act as a translation enhancer in yeast. However, as the earlier mutational analysis demonstrated (Figs. 2, 3), the AUG context on its own is not sufficient to explain the activation of translation by BBV-seq, and other, more distal nucleotide positions are important for the full activation potential of this leader sequence.

### BBV-seq functions as an eIF4G1-dependent CITE element during translation initiation

**Figure 7:**
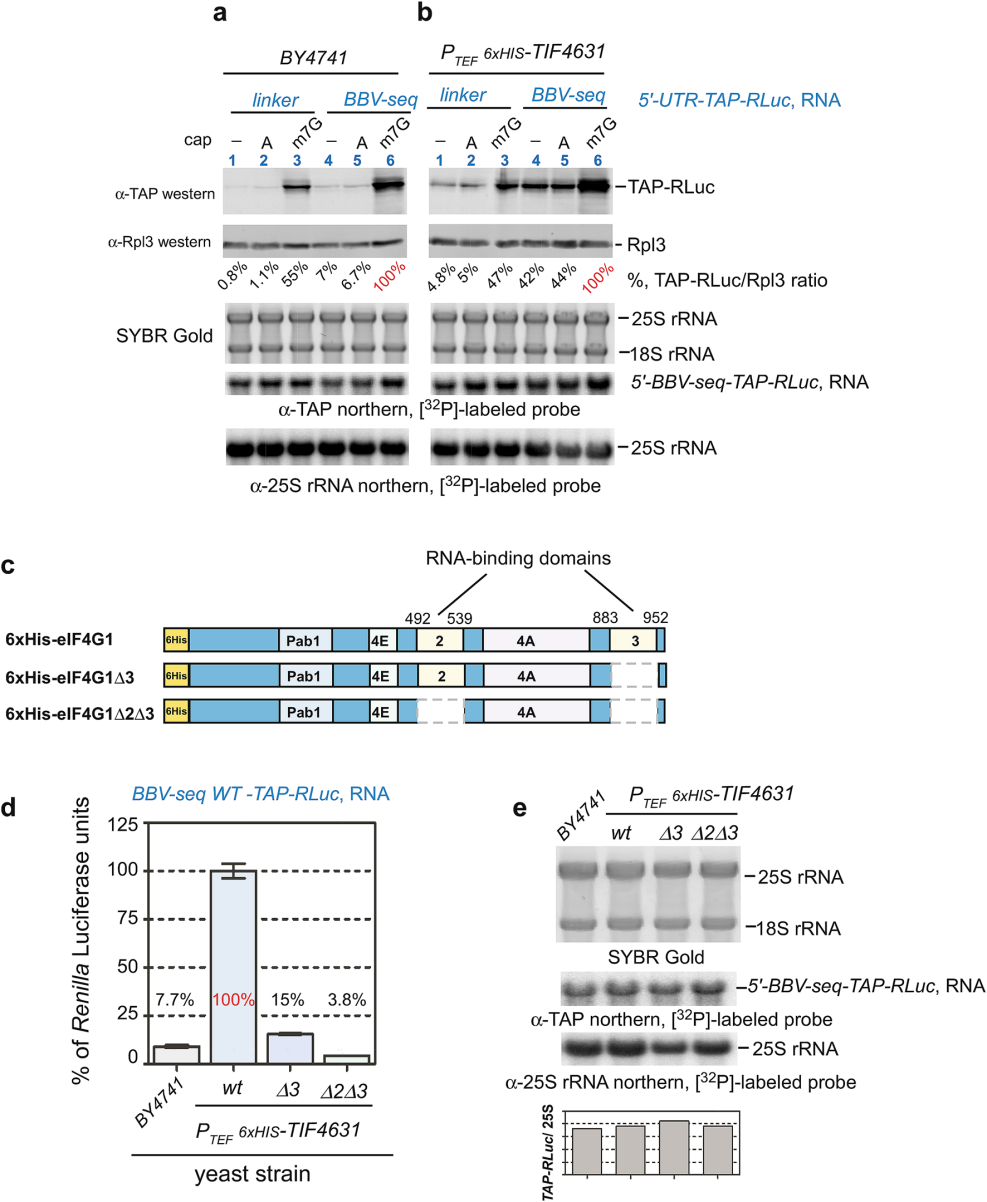
BBV-seq-mediated translation depends on RNA-binding domains of eIF4G1. (**a**,**b**) Linker- or BBV-seq-containing mRNAs were capped in vitro with m7G-cap (m7G), ApppG-cap (A), or remained uncapped (−), and were translated in yeast lysates prepared from the wild-type BY4741 strain (**a**) or from the *P*_*TEF*_*-6*× *HIS-TIF4631* strain, which overexpresses eIF4G1 (**b**). Translation products were analyzed by quantitative western blotting as described in “Methods” section (top). Quantification and normalization of TAP-RLuc are shown in Supplementary Fig. S5; TAP-RLuc amount in the reaction charged with m7G-capped *BBV-seq-TAP-RLuc* RNA was set as 100% (shown in red) to calculate the relative % of the reporter produced in each reaction. RNA was extracted from the same translation reactions, resolved on agarose gel under denaturing conditions, and stained with SYBR Gold prior to transfer, followed by northern hybridizations with the indicated probes (bottom). (**c**) Schematic representation of major domains in yeast eIF4G1. Pab1 indicates the poly(A)-binding protein domain, 4E is the eIF4E-binding domain, 4A is the eIF4A-binding domain, the RNA-binding domains are also shown. A 6× His tag was placed at the N-terminus of eIF4G1 (marked in yellow). 6× His-eIF4G1 indicates full-length protein; 6× His-eIF4G1Δ3 and 6× His-eIF4G1Δ2Δ3 designate proteins with RNA-binding domain 3 or 2/3 deletions. Numbering on the top indicates amino acid positions in eIF4G1. (**d**) Translationally competent yeast lysates indicated on the figure were programmed with *BBV-seq-TAP-RLuc* mRNA. Protein translation was assessed using *Renilla* luciferase assays and is presented as bar graphs. Data are represented as % *Renilla* Luciferase units derived from the reactions performed with lysate from *P*_*TEF*_*-6*× *HIS-TIF4631* cells programmed with *BBV-seq-WT-TAP-RLuc* mRNA. Error bars represent standard error of the mean (SEM) of three individual experiments. (**e**) RNA was extracted from luciferase reactions in (**d**) and analyzed by SYBR Gold staining, followed by northern hybridizations with the indicated probes. The *TAP-RLuc*/25S rRNA ratio for each reaction is shown as a bar graph.

One technical limitation of in vitro capping (Fig. 6a) is that some transcripts in the reaction may remain uncapped or be capped incorrectly, in which case the BBV-seq could potentially function as a CITE. To test this hypothesis, we examined whether 5′-end modification of the *BBV-seq*-containing RNA with ApppG-cap (A-cap) would influence BBV-seq-mediated translation. In fact, one defining feature of a CITE is functionality on uncapped or A-capped mRNAs^30,31^. As a control, we used RNA modified with a conventional m7G-cap. Consistent with our previous results (Figs. 5, 6a,b), synthesis of TAP-RLuc was enhanced by m7G-capping of mRNAs, and stimulation by BBV-seq and the m7G-cap was additive (Fig. 7a, lanes 3 and 6; see Supplementary Fig. S5). The presence of BBV-seq enhanced TAP-RLuc translation from both uncapped and A-capped mRNAs (Fig. 7a; see Supplementary Fig. S5). A comparable increase in the reporter protein production was not observed when mRNA containing cap-dependent 5′ UTRs from tobacco mosaic virus was used^55,56^ (see Supplementary Fig. S6). The m7G-capped, A-capped, and uncapped RNAs tested in this experiment remained stable in the translation reaction, as determined by northern hybridizations (Fig. 7a; see Supplementary Figs. S5, S6).

To initiate translation, certain CITE elements require increased levels of the translation initiation factor eIF4G1^57^, which acts as a scaffolding protein during canonical cap-dependent initiation, but can also operate in unconventional translation initiation mechanisms^2,4,5^. Furthermore, yeast eIF4G1 was found to be a limiting factor in cap-independent translation initiation^27^. This prompted us to examine whether an increased level of eIF4G1 expression could enhance protein synthesis when the BBV-seq-containing transcript is uncapped or A-capped. We generated a strain of *S. cerevisiae* in which *TIF4631* (the gene encoding eIF4G1) was placed under the control of the strong constitutive *TEF* promoter and integrated into the chromosomal HO locus. The incorporation of an extra copy of *TIF4631* was verified by sequencing the resulting hybrid HO locus, and expression of the protein was further confirmed by western blotting using antibodies against the 6× His-tag fused to the N-terminus of the HO-integrated eIF4G1 (see Supplementary Fig. S7). Although the predicted molecular weight of eIF4G1 is 107 kDa, 6× His-eIF4G1 migrated on SDS–polyacrylamide gel as ~ 150 kDa (see Supplementary Fig. S7), in line with the previous study^58^. Next, we prepared translationally active yeast lysates from the newly generated *P*_*TEF*_*-6*× *HIS-TIF4631* strain and charged this lysate with the uncapped, m7G-capped and A-capped *BBV-seq-* and linker control *TAP-Rluc* RNAs. We found that extra copies of eIF4G1 enhanced the expression of TAP-RLuc from BBV-seq 5′ UTR (Fig. 7b, lanes 4–6; see Supplementary Fig. S5). In particular, translation of uncapped or A-capped *BBV-seq-TAP-RLuc* RNA in *P*_*TEF*_*6xHIS-TIF4631* reached almost 50% of the level of capped *BBV-seq-TAP-RLuc* in the same extract (Fig. 7b, lanes 4–6; see Supplementary Fig. S5). This is a significant change in comparison with relative levels of BBV-mediated translation in wild-type yeast lysates, in which TAP-RLuc generated from uncapped/A-capped RNA constituted only 7% of the products generated from m7G-capped *BBV-seq-TAP-RLuc* RNA (Fig. 7a, lanes 4–6; see Supplementary Fig. S5).

Next, we charged the wild-type and *P*_*TEF*_*-6*× *HIS-TIF4631* lysates with *BBV-seq-TAP-RLuc* RNA. Remarkably, *Renilla* luciferase activity increased ~ 13-fold with lysates from cells expressing 6× His-eIF4G1 from the *TEF* promoter (Fig. 7d, compare bars 1 and 2; see Supplementary Fig. S7). mRNA was stable in both extracts tested (Fig. 7e, lanes 1 and 2). These data suggest that eIF4G1 indeed participates in BBV-seq-mediated, cap-independent translation initiation.

### RNA-binding domains of the translation initiation factor eIF4G1 potentiate BBV-seq-dependent translation initiation

Yeast eIF4G1 contains multiple domains important for coordinating the assembly of the translation initiation machinery (Fig. 7c). Previous work conducted with eIF4G1 from *Saccharomyces cerevisiae* established the ability of eIF4G1 to interact with RNA in vitro^58^. To test RNA-binding domains of eIF4G1 identified in that study, we deleted sequences corresponding to amino acids 492–539 (RNA-binding domain 2) and 883–952 (RNA-binding domain 3) in *TIF4631* integrated into the HO locus (schematic in Fig. 7c). As before, we verified correct mutations by sequencing the genomic locus and confirmed the expression of 6× His-eIF4G1∆3 and 6× His-eIF4G1∆2∆3 by western blotting using anti-6× His antibodies (see Supplementary Fig. S7). Similarly to eIF4G1, the two generated eIF4G1 mutants also demonstrated anomalous migration on SDS–polyacrylamide gels, likely due to deletion of positively charged arginine-rich domains and change in the net charge of the mutant proteins^59^.

Using this experimental platform, we next tested production of TAP-RLuc from the BBV-seq 5ʹ-leader in the cell-free translation reactions reconstituted with lysates from the wild-type strain and strains that expressed extra copies of eIF4G1, with or without its RNA-binding domains. Compared to the strain expressing full-length 6× His-eIF4G1, we observed a ~ 6.5-fold decrease in luciferase activity with cell extracts derived from the 6× His-eIF4G1∆3 strain. This effect was exacerbated (up to a 26-fold decrease) by the additional deletion of the RNA-binding domain 2 in the *6*× *His-eIF4G1∆2∆3*-expressing strain (Fig. 7d, compare bars 2, 3 and 4; see Supplementary Fig. S7). Northern hybridization after completion of translation showed that the stability of mRNAs in these different strains was similar (Fig. 7e; see Supplementary Fig. S7).

Together, these data support a mechanistic model in which BBV-seq-mediated translation initiation on uncapped mRNA involves recruitment of eIF4G1 to BBV-seq, likely via the RNA-binding domains of this factor.

## Discussion

**Figure 8:**
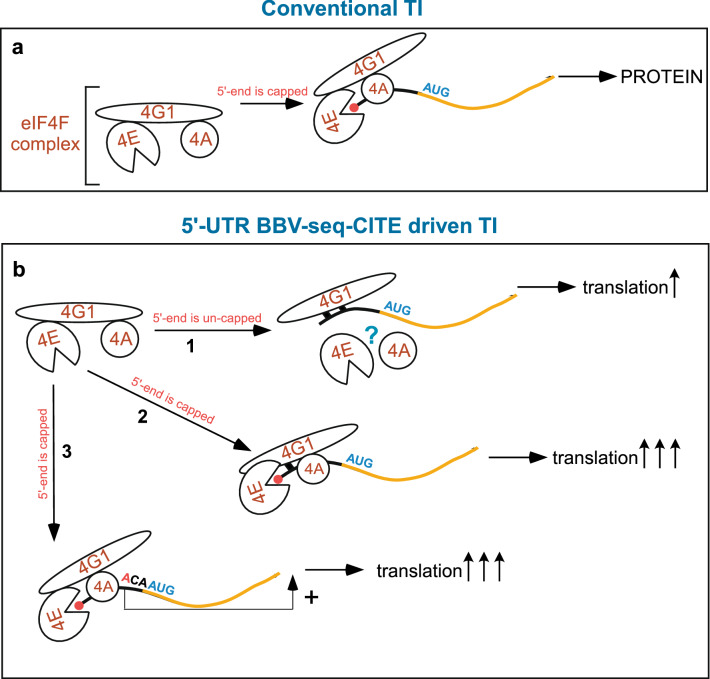
Models for conventional, cap-dependent, and proposed BBV-seq-CITE mediated translation initiation. (**a**) During conventional translation initiation (TI), the eIF4F complex (shown on the left) primes cap-modified mRNA (cap is shown as red circle). eIF4E binds to the m7G cap, while eIF4G1 acts as a scaffolding protein by placing the helicase eIF4A on the 5ʹ-UTR (black line). eIF4F activity promotes efficient translation initiation, resulting in decoding of mRNA (orange line) and efficient protein synthesis ($\uparrow \uparrow \uparrow )$. (**b**) During BBV-seq-CITE-driven translation initiation, (1) eIF4G1 interacts with uncapped BBV-seq RNA via the RNA-binding domains (shown as black squares), ultimately leading to protein synthesis ($\uparrow )$. When mRNA is capped (2, 3), translation initiation occurs via the conventional mechanism described in (a), and likely simultaneously with a direct BBV-seq-mediated recruitment of eIF4G1 (2); whereby BBV-seq also acts as an enhancer element (3) resulting in efficient protein synthesis ($\uparrow \uparrow \uparrow )$.

Initiation of protein translation is a complex and rate-limiting process that involves multiple *cis*-acting RNA elements and *trans-*acting factors, ultimately resulting in the formation of elongation-competent 80S ribosomes positioned on the initiation codon of an mRNA^4^. During conventional translation initiation in eukaryotes, the eIF4F complex is recruited to the 5ʹ-UTR of mRNA prior to other steps of translation initiation. The eIF4E subunit of the eIF4F complex binds the m7G cap-modified 5ʹ-end of a transcript, while eIF4G interaction promotes recruitment of the RNA-helicase eIF4A to the mRNA. eIF4A unravels the secondary structure of the leader sequence, facilitating mRNA scanning by 43S PIC in search of the start codon. In this mechanism, eIF4G acts as a scaffolding protein (Fig. 8a). Results from our study suggest a different mechanism of translation initiation governed by an uncapped, 39-nucleotide BBV-seq 5ʹ-UTR^39^ (Fig. 1b), which operates using eIF4G1 and depends on RNA-binding domains of this initiation factor (Fig. 8b-1).

Using a yeast cell-free translation system, we detected robust protein production with uncapped TAP-*Renilla* luciferase (TAP-RLuc) reporter mRNAs when BBV-seq was placed upstream of the reporter ORF (Fig. 1). Expression levels of the TAP-RLuc controlled by BBV-seq were comparable to those driven by several known IRES elements (Fig. 1d). Unlike in many viral IRESs, only weak secondary structures are predicted in BBV-seq (see Supplementary Fig. S2, Supplementary Tables S1, S2), arguing that this insect virus-derived sequence lacks extensive RNA folding. Given that certain IRES elements are unstructured^26,27,49^, we conducted a series of experiments to prove that BBV-seq does not promote internal ribosome entry but operates by a scanning mechanism during translation initiation. As such, we found no expression of ORF2 (*Rluc*) from the bicistronic reporter construct TAP-BBV-seq-RLuc (Fig. 5). These results are in accord with the negligible reporter protein expression observed when the 5′-end of RNA was modified with a hairpin (Fig. 4b,c). These biochemical approaches support the conclusion that the 39-nt BBV-seq examined here is not an IRES.

Having established that BBV-seq does not function as an IRES but rather relies on its sequence, we studied the sequence requirements for efficient translation initiation using mutational analysis. We found that BBV-seq is required in its entirety for efficient translation initiation (Fig. 3). At the same time, the free 5′ end of mRNA (Figs. 4, 7a) and positioning of the start codon in the optimal context provided by the 3′ end of BBV-seq (Fig. 6c–e) are both essential for the activation of cap-independent translation by this sequence.

Importantly, genetic evidence suggests that BBV-seq-mediated translation depends on RNA-binding domains of the translation initiation factor eIF4G1 (Fig. 7c–e, Supplementary Fig. S7). Previous studies conducted in yeast and mammalian cells have revealed that 5ʹ-UTRs of uncapped transcripts can interact with eIF4G1 directly or indirectly. As such, mRNAs encoding the set of yeast proteins responsible for pseudohyphal growth (a cellular stress response to limited nutrients) contain A-rich 5ʹ-UTRs that directly interact with poly-A binding protein (Pab1/PABP), which recruits eIF4G1, thus overcoming the dependence on cap and cap-binding protein eIF4E^27^. According to another model, mammalian eIF4G1 directly and specifically binds leader sequences of HIF-1α, FGF-9, and p53 mRNAs; this is thought to play a pivotal role during the stress response when capping is blocked^1^. These data were further supported by the studies demonstrating that eIF4G can indeed directly contact 5ʹ-UTRs and promote translation initiation in a cap-independent manner^1,30,60,61^. For example, eIF4G can bind to domain V of the type I IRES elements of coxsackievirus B3, enterovirus 71, and poliovirus^60,61^, to the type II encephalomyocarditis virus (EMCV) IRES; and to β-globin mRNA^62,63^. Thus, it seemed possible that eIF4G1 may directly interact with RNA leader sequences, the translation of which occurs via cap-independent mechanisms, constituting a prerequisite step for recruitment of other factors needed for translation initiation. This model predicts that Arg-rich RNA-binding domains that can bind RNA directly^58^ may promote eIF4G1 interaction with BBV-seq. RNA-binding domain(s)-deleted strains are translationally impaired, which is somewhat difficult to address in cellular systems. However, the generated *P*_*TEF*_*-6*× *HIS-TIF4631* strain allowed us to overcome this experimental obstacle. First, *P*_*TEF*_*-6*× *HIS-TIF4631* demonstrates increased translational efficiency over the wild-type strain (Fig. 7d, bars 1 and 2) and, thus, intensifies any potential differences in translation of reporter mRNAs. Secondly, *P*_*TEF*_*-6*× *HIS-TIF4631* is viable due to the presence of endogenous *TIF4631*, allowing preparation of yeast lysates to evaluate the impact of RNA-binding domains on BBV-seq-driven translation.

Using this strain, we addressed whether initiation of the BBV-seq depends on eIF4G1’s RNA-binding domains. We found that eIF4G1∆3 maintains residual activity and augments the BBV-seq-mediated translation initiation mediated by the endogenous factor (Fig. 7d). However, deletion of RNA-binding domains 2 and 3 on the *PTEF-6* × *HIS-TIF4631* background resulted in a severe decrease in protein production in cell-free translation reactions and suppresses endogenous eIF4G1 (Fig. 7d), implying that the eIF4G1Δ2Δ3 acts as a dominant-negative inhibitor. These data are consistent with the genetic data, whereby a single deletion mutant of RNA-binding domain 3 was temperature-sensitive, while dual deletion of the domains 2 and 3 (Δ2Δ3) was lethal^58^, underscoring the important role RNA binding plays in eIF4G1 activity. One possible mechanism for the dominant negative activity of the eIF4G1Δ2Δ3 mutant is through sequestering the eIF4A helicase, as (i) eIF4G1 and eIF4A interact directly^64^, (ii) eIF4G1’s RNA-binding domains 2 and 3 do not overlap with its eIF4A-binding domain (Fig. 7c) and ^58^, and (iii) besides unraveling RNA structure, eIF4A was implicated in providing directionality to the 43S complex during scanning, thereby decreasing the average dwell time of 43S on the 5′-UTR^49^. Another possibility might be that yeast eIF4G1 binds leader RNA via its HEAT domain similarly to mammalian eIF4G^65,66^, while the arginine-rich sequences previously annotated as RNA-binding domains 2 and 3 (examined here) may confer correct protein conformation. In this scenario, eIF4G1Δ2Δ3 might bind BBV-seq via the HEAT domain but in an unproductive fashion, competing with endogenous wild-type eIF4G1 for interactions with 5′ UTR. In fact, mammalian eIF4G’s HEAT domain binds specifically to EMCV and other picornavirus IRESs^65^ as well as to eIF4A, and an adjacent domain binds to β-globin mRNA and has been implicated in scanning^62,63^. Future experiments are needed to distinguish between these two models by exploring details of eIF4G1 binding to BBV-seq RNA. Regardless, the results presented here support a model in which eIF4G1 captures BBV-seq RNA, facilitating 43S PIC recruitment and translation initiation (Fig. 8b-1). It will also be of interest to investigate the participation of eIF4G2 in the process of cap-independent translation, considering that this factor is known to interact with mRNA and UTRs^67^, while mammalian eIF4G2/DAP5 binds CITE elements identified for several mRNAs^1^. Another focus of the future studies should be the investigation of BBV-seq-mediated cap-independent translation initiation using poly-adenylated mRNA to determine whether PABP contributes to this eIF4G1-operated mechanism.

One other interesting property of BBV-seq is its ability to enhance cap-dependent translation initiation (Fig. 6a,b). One explanation of this result is that cap-dependent and BBV-seq-mediated cap-independent mechanisms may act simultaneously (Fig. 8b-2). Given that the RNA-binding domain 2 of eIF4G1 is located between the eIF4E- and eIF4A-binding domains^57,64^, it is possible that in a cap-eIF4E-eIF4G1 chain of interactions, cap-proximal mRNA may form a direct contact with eIF4G1, thus increasing the efficiency of initiation. Furthermore, our mutational analysis has revealed strong dependency on the adenine residue present at position − 3 relative to the AUG start codon on translational efficiency governed by BBV-seq. This discovery resembles characteristics of the Kozak sequence^15,16,46,68^, suggesting that besides an ability to initiate translation of uncapped mRNAs, BBV-seq acts as a regulatory sequence and may participate in selection of the correct codon (Fig. 8b-3). These intriguing models are subjects of further investigation.

Terenin et al*.* analyzed translation of mRNA placed under the control of only a portion of the EMCV-IRES (J-K domains)^30^. It was found that EMCV-J-K leader sequence acts as a CITE element that directly binds eIF4G and promotes translation initiation by a mechanism that requires a free 5′-end and involves ribosomal scanning. Thus, the CITE mechanism is distinct from IRES-mediated translation initiation, as it depends on 5ʹ-end availability and functions upon modification of the 5′-end with an A-cap. In many cases CITEs do not require a full set of translational factors^31^. Finally, a recent study conducted in mammalian cells has revealed the requirement for a single-stranded and unstructured A-rich sequence for cap-independent translation initiation^69^. All these characteristics of CITE are inherent to BBV-seq dissected in this work (Figs. 4, 5, 7a,b, Supplementary Fig. S6). Therefore, we propose that BBV-seq sequence constitutes a previously undescribed CITE element that in yeast recruits eIF4G1 for efficient binding of 43S PIC and positioning the 40S subunit for scanning to identify the start codon of the downstream gene (Fig. 8b-1).

From the practical viewpoint, the small size of BBV-seq makes it an attractive biological tool for protein translation applications in heterologous settings. By incorporating this sequence in a forward primer during amplification of the gene of interest (as shown in Fig. 1a), translation efficiency can be increased, and further improved by using yeast strains expressing extra copies of eIF4G1 (Fig. 7d). Thus, using the *P*_*TEF*_*-6* × *HIS-TIF4631* strain generated in this study may aid in applications using uncapped RNA transcripts with the translationally competent cell-free yeast reaction system.

## Methods

Yeast strains and growth medium; antibodies, chemicals and enzymes used in this study; plasmids and their generation are described in detail in Supplementary Methods.

### PCR and RNA preparation

PCR reactions were performed with DreamTaq (Thermo Fisher) in 50 μL reaction volume using 10 ng of a pYes plasmid construct as a template, 0.4 μM forward (5′-CGGATCGGACTACTAGCAGCTG -3′) and 0.4 μM reverse (5′-TTCATTAATGCAGGGCCGCAAATT-3′) primers that anneal upstream and downstream of the *P*_*T7*_ and the *TER*_*CYC1*_ elements, respectively. PCR products were purified and concentrated using type D4004 ZYMO column (ZYMO Research) and DNA concentrations were estimated spectrophotometrically. To synthetize uncapped RNA, 1 μg of PCR-generated DNA template was transcribed in the T7-polymerase reaction using TranscriptAid T7 High Yield Transcription kit (Thermo Scientific) according to the manufacturer's recommendations. Reaction mixtures were incubated for 120 min at 37 °C, followed by DNase treatment for 15 min at 37 °C. RNA transcripts were first purified by phenol/chloroform extraction, followed by EtOH precipitation; pellets were resuspended in 50 μL of H_2_O and separated from unincorporated nucleotides by gel filtration on Micro Bio-Spin P-30 columns (Bio-Rad). RNA was aliquoted and stored at − 80 °C.

To generate capped RNA, purified PCR products (described above) were used as DNA templates in coupled T7 RNA polymerization–RNA capping reactions (mMESSAGE mMACHINE kit, Thermo Fisher #AM1344M), incubated for 1 h at 37 °C.

### Structural modeling and RNA folding analyses

We predicted putative secondary structures and their respective ΔG values (kcal/mol) for wild type or mutant RNA sequences using the "Predict a Secondary Structure” server (RNA structure tools version 6.0.1) (https://rna.urmc.rochester.edu/RNAstructureWeb/).

### Northern blotting and signal quantification

RNA was separated on 1.2% formaldehyde-containing agarose gel as described previously^70^. Gels were stained with SYBR Gold (Invitrogen) and scanned using a Typhoon 9200 imager (GE Healthcare) at 532 nm to visualize RNA. RNA was then transferred onto nylon membrane and hybridized with a [^32^P]-labeled probe specific for the gene encoding TAP (5′-GCCGAATTCTCCCTGAAAA-3′) or with [^32^P]-labeled probe y540 against 25S rRNA (5′-TCCTACCTGATTTGAGGTCAAAC). Radioactive signal was detected using Typhoon 9200 in the phosphorimaging mode and analyzed using ImageQuant software (GE Healthcare). For quantification, volume of the hybridization signal corresponding to the RNA species of interest was converted to phosphorimaging units, and the background (average image background) was subtracted. For normalization of *TAP* RNA, the phosphorimaging units corresponding to this RNA were divided by that derived from the 25S rRNA signal present in the same sample.

### Preparation of translationally active lysates

*Saccharomyces cerevisiae* cells grown in 1 L of YPD medium to OD_600_ ~ 0.5 were harvested, washed, incubated at room temperature in 1 M sorbitol, then in YPD. Cells were collected, washed in buffer A (20 mM Hepes–KOH (pH 7.4), 100 mM KOAc, 2 mM Mg(OAc)_2_, 2 mM DTT) and the pellet was resuspended in buffer A containing 25% mannitol and 0.1 mM PMSF in 2:3 volume/cell weight ratio. The cell slurry was dripped directly into liquid nitrogen to form small ice beads. Frozen yeast/buffer beads were powdered in a SPEX freezer mill chamber. The powdered yeast cells were thawed and centrifuged in a Beckman ultracentrifuge for 15 min at 30,000×*g* using a fixed angle Beckman rotor Type 80 Ti. The clear phase was collected (~ 4 mL), centrifuged for 30 min at 100,000×*g* using the same rotor and the supernatant was collected. 2.5 ml of supernatant was applied on gel filtration columns G-25 (GE Healthcare) and 5 mL of buffer A containing 10% glycerol was used for elution. The RNA content in eluted fractions was determined spectrophotometrically; fractions with at least 75% of the highest RNA concentration were pooled, aliquoted and frozen in liquid nitrogen for storage at − 80 °C.

### Cell-free yeast translation reactions

The translation reactions were carried out as described previously^43^, with a few modifications. In brief, for one reaction we used 7.5 μL of yeast extract (final protein concentration ~ 25–30 mg/mL), 2 μL of RNA (100–300 ng), 3 μL of 5 × translation buffer (100 mM Hepes–KOH (pH 7.6), 50 μM each amino acid, 10 mM Mg(OAc)_2_, 250 mM KOAc, 100 mM creatine phosphate, 0.6 U/μL creatine phosphokinase, 10 mM DTT, 5 mM ATP, 0.5 mM GTP, 15 U RiboLock, 5× SIGMAFAST Protease Inhibitor Cocktail), 2.5 μL of H_2_O. The total reaction volume was 15 μL. Reactions were incubated at 21 °C for 90 min. Prior to reaction, RNA was folded. The folding reactions containing 10 μg of mRNA transcript in 12 μL of H_2_O were heated at 95 °C for 2 min, cooled on ice for 2 min, mixed with 6.6 μL folding buffer (333 mM HEPES, pH 8.0, 20 mM MgCl_2_, 333 mM NaCl) and incubated at 37 °C for 30 min^71^. We found no differences in protein yield between folded or unfolded mRNA containing BBV-seq; therefore, we omitted the RNA folding step when BBV-seq-containing RNAs were included in cell-free translation reactions.

### Western blotting and signal quantification

To analyze in vitro translation reaction products, proteins were isolated from 15 μL of the translation reactions using TRI REAGENT-LS according to the manufacturer’s recommendations. Protein pellets were resuspended in 50 μL of 1xSDS-PAGE loading dye, and 10 μL were analyzed by SDS-PAGE using 10% polyacrylamide gels in duplicate. To analyze expression of 6xHis-eIF4G1 and its RNA-binding domain deletion mutants, the corresponding yeast strains were lysed in Buffer A supplemented with PMSF and SIGMAFAST Protease Inhibitor Cocktail by glass-beads shearing. The lysates were clarified by centrifugation and a total of 10 μg protein, as measured by the Bradford assay, was separated by SDS-PAGE using 8% polyacrylamide gels. Proteins were transferred onto nitrocellulose membrane and Ponceau S stained to verify equal loading. For conventional western blotting with ECL detection, we used 10% milk in PBS—0.1% Tween 20 (PBST) for blocking (20 min), and PBST for washing. For quantitative western blotting, Ponceau S staining was omitted; prior to blocking, membranes were dried at 37 °C for 15 min, rehydrated and blocked with 5% milk in PBS for 60 min. For detection, membranes were dried at 37 °C for 30 min and kept in a dark chamber. We used IR-Dye-680RD secondary antibodies for Rpl3-probed blots, and IR-Dye-800CW antibodies for TAP-probed blots. IR signals were detected on a Typhoon imager by scanning membranes at 800 nm and 680 nm; ImageQuant was used to analyze the images. For normalization, bands corresponding to TAP-RLuc and Rpl3 were converted to phosphorimager units and TAP-RLuc/Rpl3 ratios were plotted as bar graphs.

### Renilla luciferase assays and statistical analysis

We used the *Renilla* Luciferase Assay System from Promega (cat#E2810) according to the manufacturer's protocol. The luminescent signal was measured on a GLOMAX 20/20 luminometer. Statistical analysis was performed by one-way ANOVA with GraphPad PRISM 5.

For RNA and protein extraction from the luciferase reaction, 100 μL of TRI REAGENT-LS reagent were added to 100 μL of the luciferase reaction. Samples were stored at − 80 °C prior to processing according to the manufacturer’s recommendations.

## Supplementary Information

Supplementary Information.
