# Supplementary material for: A translation enhancer element from black beetle virus engages yeast eIF4G1 to drive cap-independent translation initiation
Source: Sci Rep. 2021 Jan 28;11:2461. doi: 10.1038/s41598-021-82025-6 (PMC7844027; doi:10.1038/s41598-021-82025-6)
Supplement: Supplementary file 1 — Supplementary Information. [file 41598_2021_82025_MOESM1_ESM.pdf]

SUPPLEMENTARY INFORMATION

A translation enhancer element from Black Beetle Virus engages yeast eIF4G1 to drive cap-independent translation initiation.

Brandon M. Trainor[1,2], Arnab Ghosh[1,3], Dimitri G. Pestov[1], Christopher U. T. Hellen[4] and Natalia Shcherbik[1*]

[1] Department of Cell Biology and Neuroscience, Rowan University, School of Osteopathic Medicine, 2 Medical Center Drive, Stratford, NJ 08084, USA

[2] Graduate School of Biomedical Sciences, Rowan University, 42 E. Laurel Road, Suite 2200, Stratford, NJ 08084, USA

[3]Current address: Center for Gene Regulation in Health and Disease, Cleveland State University, 2121 Euclid Ave, Cleveland, OH 44115, USA

[4]Department of Cell Biology, State University of New York Downstate Health Sciences University, 450 Clarkson Avenue MSC 44, Brooklyn, New York 11203

*E-mail: shcherna@rowan.edu

Natalia Shcherbik, PhD
Assistant Professor
Department of Cell Biology,
School of Osteopathic Medicine
Rowan University
2 Medical Center Drive, Stratford NJ 08084-1489
Phone: 856-566-6914

SUPPLEMENTARY INFORMATION

Supplementary Methods (page 2-5)

Table S1-S3 (page 6-8)

Figure S1 (page 9-10)

Figure S2 (page 11-12)

Figure S3 (page 13)

Figure S4 (page 14)

Figure S5 (page 15)

Figure S6 (page 16-17)

Figure S7 (page 18-19)

The source files (page 20-27)

Supplementary methods

Growth medium and yeast strains.

We used standard YPD medium (1% yeast extract, 2% peptone and 2% dextrose) sterilized by filtration through 0.2 μm PES filters (Thermo Scientific). Wild-type *S. cerevisiae* BY4741 (*MAT***a** *his3–1 leu2– 0 met15– 0 ura3– 0*) was obtained from Open Biosystems. To generate the *P_{TEF}-6×HIS-TIF4631* strain, a *KanMX-P_{TEF}-6×HIS-TIF4631-T_{CYC1}* cassette was generated by PCR with primers designed to add flanking HO homology arms from the integration plasmid construct pTEF:*6×HisTIF4631* (see below). Genomic DNA was isolated from G418-resistant colonies of BY4741 cells appearing after transformation with the above cassette and verified for the correct integration into the HO locus by PCR using a primer annealing ~100 bp upstream of the integration site and a second primer annealing within the KanMX cassette. To generate mutant derivatives of the *P_{TEF}-6×HIS-TIF4631* strain, designated as *P_{TEF}-6×HIS-TIF4631Δ3* and *P_{TEF}-6×HIS-TIF4631Δ2Δ3*, we used similar manipulations as for *P_{TEF}-6×HIS-TIF4631* strain generation.

Antibodies, chemicals and enzymes.

We used the following antibodies: anti-TAP (Invitrogen, # CAB 1001); anti-Rpl3 (ScRPL3, Developmental Studies Hybridoma Bank, University of Iowa); anti-mouse 6xHis antibodies fused with HRP (Rockland, cat# 200-303-382); anti-mouse IgG-HRP (GE-Healthcare, Cat# NA931); anti-rabbit IR-Dye 800CW, (LI-COR, Cat# 925-32211); anti-mouse IR-Dye 680RD, (LI-COR, Cat# 925-68070).

G418 was purchased from Thermo Fisher Scientific, TRI REAGENT-LS was from Molecular Research Center (cat # TS 120), ECL substrate was from Millipore; DTT was purchased from Sigma, PMSF was from Amresco; creatine phosphate was from Alfa Aesar.

RiboLock was purchased from Thermo Fisher; SIGMAFAST™ Protease Inhibitor Cocktail was from Sigma; all the restriction enzymes and polynucleotide kinase (PNK) were obtained from Thermo Fisher. Creatine phosphokinase was purchased from BioVision.

Plasmids.

pYes was purchased from Invitrogen. To generate the initial construct **pYes-TAP-RLuc**, we used a two-step cloning strategy. First, we amplified the TAP sequence using the pBS1761 plasmid as a template with the forward primer containing a BamHI site and the reverse primer containing an XhoI site, whereby the stop codon was omitted from the coding sequence on the reverse primer. The amplified TAP sequence was cloned into pYes between BamHI and XhoI sites. The *Renilla* luciferase gene amplified by PCR from pJD375 using a forward primer containing an XhoI site and a reverse primer containing an XbaI site was cloned into pYes-TAP between XhoI and XbaI sites. The generated pYes-TAP-RLuc construct was used as the "linker sequence" control in this study, that we termed "(-1) pyrimidine linker".

To generate pYes-CrPV-TAP-RLuc, the CrPV sequence was amplified by PCR from the dicistronic Rluc-CrPV IGR-FLuc (Wilson et al., 2000) using a HindIII-containing forward primer and a BamHI-containing reverse primer and cloned between HindIII and BamHI sites of pYes-TAP-RLuc. Sequences for URE2, NCE102 and YMR181c were generated by Genewiz and contained HindIII and BamHI sites at the 5′- and 3′-ends, respectively. These double-stranded DNA fragments were individually ligated with pYes-TAP-RLuc digested with HindIII and BamHI.

The original pYes-BBV-seq-TAP-RLuc construct was generated as follows: two BBV sequence oligonucleotides (top and complementary bottom strands) were synthesized by Sigma-Genosys; 100 pmoles of each oligo were phosphorylated by PNK in the presence of 10 mM ATP for 30 min at 30°C, oligos were combined, heated at 80°C for 10 min and slowly cooled down. After annealing, HindIII and BamHI sticky ends were formed at the 5′-end and 3′-end of the BBV-seq insert, respectively. This insert was ligated into pYes-TAP-RLuc that had been digested with HindIII and BamHI enzymes.

We also used a different strategy to generate the pYes-BBV-seq-TAP-RLuc* construct (shown in Figure 1C) and its derivatives, based on incorporation of BBV-seq wild-type or mutant sequence(s) into the forward primer. This primer also contained a BamHI site immediately upstream of the BBV-seq sequence or its derivatives and a region complementary to the first 28 nucleotides within the TAP gene sequence (see Table S3). The reverse primer contained an XbaI site and a region complementary to the 3′-end of the *Renilla* luciferase gene. Thus, the PCR products carried BBV-seq (wild-type or mutant) followed by the TAP-RLuc gene sequence; they were digested with BamHI and XbaI and ligated with pYes cleaved with BamHI/XbaI. The resulting pYes-BBV-seq-TAP-RLuc* construct has HindIII, KpnI and BamHI sites available. The pYes-(-1) purine-TAP-RLuc and pYes-unstructured linker-TAP-RLuc constructs were generated using similar strategy, wherein we amplified TAP-RLuc sequence using the forward primer that contained either sequence of (-1) purine linker or sequence of unstructured linker (see Table S3) and HindIII site, while the reverse primer contained XbaI site and a region complementary to the 3′-end of the *Renilla* luciferase gene. PCR products were digested with HindIII and Xba1 and ligated with pYes between HindIII/Xba1 sites.

To generate pYes-Hairpin-BBV-seq-TAP-RLuc, two oligonucleotides were synthesized by Sigma-Genosys: 5′-AGCTGATCCTAGGATCCTAGGATCCTAGGATCCTAG-3′ and 5′-GATCCTAGGATCCTAGGATCCTAGGATCCTAGGATC-3′. The oligos were phosphorylated and annealed to each other as described above. Annealing resulted in formation of HindIII and BamHI sites at the 5′-end and 3′-end of the double-stranded DNA fragment, respectively. The hairpin DNA fragment was then cloned into pYes-BBV-seq-TAP-RLuc* between HindIII and BamHI sites, resulting in pYes-HP-BBV-seq-TAP-RLuc plasmid. To generate pYes-Hairpin-spacer-BBV-seq-TAP-RLuc (HS-BBV-seq-TAP-RLuc), we cloned a spacer Hairpin-15xC-BBV-seq-

TAP-RLuc sequence into pYes using forward primer that contained HindIII site followed by the hairpin-15xC sequence and a region complementary to the 5′-end of BBV-seq, while the reverse primer reverse primer contained XbaI site and a region complementary to the 3′-end of the *Renilla* luciferase gene (see Table S3). As a template, we used pYes-HP-BBV-seq-TAP-RLuc plasmid generated before.

To generate bicistronic constructs, we used three-step strategy. First, we cloned TAP sequence containing stop codon into pYes between HindIII and BamHI. TAP was amplified using primers annotated in Table S3. In parallel, we generated pYes-BBV-seq-RLuc plasmid: the BBV-seq-RLuc was amplified using the forward primer that contained BamHI site followed by BBV-seq sequence and sequence complementary to 5′-end of *Renilla* luciferase gene, while the reverse primer contained XbaI site and a region complementary to the 3′-end of the *Renilla* luciferase gene (see Table S3). PCR product was cloned into pYes between BamHI and XbaI sites. Next, we amplified 15xC-BBV-seq-RLuc using the forward primer that contained BamHI site followed by 15xC and a region complementary to the 5′-end of BBV-seq, while the reverse primer containing XbaI site and a region complementary to the 3′-end of the *Renilla* luciferase gene. As a template, we used pYes-BBV-seq-RLuc plasmid. Resulting PCR products were cloned into pYes-TAP plasmid between BamHI and XbaI sites, resulting in pYes-TAP-spacer-BBV-seq-RLuc. To generate pYes-TAP-spacer-RLuc plasmid, we amplified RLuc using the forward primer containing BamHI followed by 15xC sequence and a region complementary to 5′-end of *Renilla* luciferase gene, while the reverse primer contained XbaI site and a region complementary to the 3′-end of the *Renilla* luciferase gene (see Table S3). Finally, the hairpin oligonucleotide synthesized by Sigma-Genosys (5′- GATCCGATCCTAGGATCCTAGGATCCTAGGAT CCTAGGATCG -3′) was phosphorylated and annealed resulting in formation of BamHI sites at the 5′-end and 3′-end of the double-stranded DNA fragment. The hairpin DNA fragment was then cloned into pYes-TAP-spacer-BBV-seq-RLuc and into pYes-TAP-spacer-RLuc at BamHI site, resulting in two bicistronic constructs: pYes-TAP-HS-BBV-seq-RLuc and pYes-TAP-HS-RLuc, wherein HS stands for hairpin-spacer.

The HO locus integration plasmid pTEF:ATP was purchased from Addgene (cat #92179). To recover the insert-free backbone of the plasmid, we digested pTEF:ATP with AvrII and KpnI and purified the high molecular weight DNA fragment. *TIF4631* was amplified from genomic DNA using forward and reverse primers containing AvrII and KpnI restriction sites, respectively. A sequence encoding 6xHis was incorporated into the forward primer immediately downstream of the ATG triplet in frame with the rest of the coding sequence. The PCR product and the dephosphorylated integration vector pTEF were ligated together, yielding the pTEF:*6×HisTIF4631* construct. To generate pTEF:*6×HisTIF4631* deleted for sequences encoding RNA-binding domains 2 and 3, we amplified fragments using pTEF-*6×His-TIF4631* as a template with the following primers: 5′-ATAGCAATCTAATCTAAGTTTCCTAGG-3′ and 5′-TCTATTTCCTCTGCCCATTCCTG-3′; and 5′-CAGGAATGGGCAGAGGAAATAGAA

ATAGATCTTATACATCAAGAAGAGAC-3′ and 5′-GAGCGGATCTTAGCTAGCCGCGG TACCTTATCTTGAATTATTCTTCAATTGACG-3′. Similarly, to generate pTEF:*6×HisTIF4631* deleted for the sequence encoding RNA-binding domains 3, we amplified the fragment using pTEF-*6×His-TIF4631* as a template with primers: 5′-ATAGCAATCTAATCTAAGTTTCCTAGG-3′ and 5′-GAGCGGATCTTAGCTAGCCGCGGTAC CTTATCTTGAATTATTCTTCAATTGACG-3′. The pTEF backbone digested with KpnI and AvrII was assembled with the PCR fragments described above using the NEBuilder® HiFi DNA Assembly Master Mix (New England Biolabs) following the manufacturer's recommendations. All constructs used in this study were confirmed by DNA sequencing (Genewiz).

Supplementary Table S1. **Gibbs free energy values (ΔG in kcal/mol) for wild-type and mutant BBV-seq sequences at 21°C and 30°C.**

BBV sequence	ΔG (21°C) kcal/mol	ΔG (30°C) kcal/mol
**BBV-seq WT**	-3.8	-2.4
**(-1) pyrimidine-linker**	-8.9	-7.6
**(-1) purine-linker**	-9.0	-7.6
**BBV-seq mutant 1**	-0.1	3.5
**BBV-seq mutant 2**	3.4	3.5
**BBV-seq mutant 3**	-0.8	-0.2
**BBV-seq mutant 4**	3.3	3.3
**BBV-seq -19/-1**	1.7	1.7
**BBV-seq -39/-13**	-2.9	-1.6

Supplementary Table S2. **ΔG values (kcal/mol) for wild-type and mutant BBV-seq sequences reflecting structural complexity of the 5'-UTR region at different temperatures.**

BBV sequence	ΔG (21°C) kcal/mol	ΔG (30°C) kcal/mol
**BBV-seq WT**	-3.8	-2.4
**BBV-seq Δ-29/-39**	2.6	2.6
**BBV-seq Δ-17/-30**	-3.4	-2.1
**BBV-seq Δ-11/-20**	-1.9	-0.8
**BBV-seq Δ-1/-10**	-3.8	-2.4

Supplementary Table S3. Sequences of primers for the generation of BBV-seq-containing constructs.

Construct	Forward primer sequence	Reverse primer sequence	Template	Related Figure
(-1) pyrimidine linker	5′**AAGCTT**GGTAACCGAGCTCGGA TCTATGAAGAGAAGATGGAAAA AGAATTTCC	5′**TCTAGA TCA**CGACATTT GTTCATTTTTGAGAACTCG-3′	pYes-TAP-RLuc	Figure S1
(-1) purine linker	5′**AAGCTT**GGTAACCGAGCTCGGA TCAATGAAGAGAAGATGGAAAA AGAATTTCC	5′**TCTAGA TCA**CGACATTT GTTCATTTTTGAGAACTCG-3′	pYes-TAP-RLuc	Figure S1
Unstructured linker	5′**AAGCTT**TGAAAGTAAAAGATGCT GAAGATCAGTTGGGTGCACGAAG **ATG**AAGAGAAGATGGAAAAAGA ATTTCC	5′**TCTAGA TCA**CGACATTT GTTCATTTTTGAGAACTCG-3′	pYes-TAP-RLuc	Figure S1
BBV-seq WT	5′**GGATCC**GTTTTCGAAACAAAT AAAACAGAAAAGCGAACCTAAACA **ATG**AAGAGAAGATGGAAAAAGAAT TTCC-3′	5′**TCTAGA TCA**CGACATTT GTTCATTTTTGAGAACTCG-3′	pYes-TAP-RLuc	Figures 1-7,S1-S7
BBV-seq reversed	5′**GGATCC**ACAAATCCAAGCG AAAAGACAAAATAAACAAAG CTTTTG **ATG**AAGAGAAGATGG AAAAAGAATTTCC-3′	5′**TCTAGA TCA**CGACATTT GTTCATTTTTGAGAACTCG-3′	pYes-TAP-RLuc	Figures 1, 2, 3, 6
BBV-seq mutant 1	5′**GGATCC**GGGAAGGAAACAAA TAAAACAGAAAAGCGAACTTAAA CA **ATG**AAGAGAAGATGGAAAAAG AATTTCC-3′	5′**TCTAGA TCA**CGACATTT GTTCATTTTTGAGAACTCG-3′	pYes-TAP-RLuc	Figures 2, S2
BBV-seq mutant 2	5′**GGATCC**GAAATTGAAACA AATAAAACAGAAAAGCGAACCTA AACA **ATG**AAGAGAAGATGGAAA AAGAATTTC-3′	5′**TCTAGA TCA**CGACATTT GTTCATTTTTGAGAACTCG-3′	pYes-TAP-RLuc	Figures 2, S2
BBV-seq mutant 3	5′**GGATCC**GGGAAGGAAACAAA TAAAACAGAAAAGCGAACCTAAA CA **ATG**AAGAGAAGATGGAAAAAG AATTTCC-3′	5′**TCTAGA TCA**CGACATTT GTTCATTTTTGAGAACTCG-3′	pYes-TAP-RLuc	Figures 2, S2
BBV-seq mutant 4	5′**GGATCC**GAAATTGAAACA AATAAAACAGAAAAGCGAACTTA AACA **ATG**AAGAGAAGATGGAAA AAGAATTTC-3′	5′**TCTAGA TCA**CGACATTT GTTCATTTTTGAGAACTCG-3′	pYes-TAP-RLuc	Figures 2, S2
BBV-seq -19/-1	5′**GGATCC**CAGAAAAGCGAA CCTAAACA **ATG**AAGAGAAGATG GAAAAAGAATTTCC-3′	5′**TCTAGA TCA**CGACATTT GTTCATTTTTGAGAACTCG-3′	pYes-TAP-RLuc	Figures 2
BBV-seq -39/-13	5′**GGATCC**GTTTTCGAAACAA ATAAAAAGAAAAG **ATG**AAGAGA AGATGGAAAAAGAATTTCC-3′	5′**TCTAGA TCA**CGACATTT GTTCATTTTTGAGAACTCG-3′	pYes-TAP-RLuc	Figures 2
BBV-seq -3A/-3C	5′**GGATCC**GTTTTCGAAACAAA TAAAACAGAAAAGCGAACCTAAC CA **ATG**AAGAGAAGATGGAAAAAG AATTTCC-3′	5′**TCTAGA TCA**CGACATTT GTTCATTTTTGAGAACTCG-3′	pYes-TAP-RLuc	Figures 6
BBV-seq -39/-31	5′**GGATCC**GTTTTCGAA **ATG**AA GAGAAGATGGAAAAAGAATTTC-3′	5′**TCTAGA TCA**CGACATTT GTTCATTTTTGAGAACTCG-3′	pYes-TAP-RLuc	Figures 3
BBV-seq -21/-29	5′**GGATCC**CAAATAAAAC **ATG**A AGAGAAGATGGAAAAAGAATTTC-3′	5′**TCTAGA TCA**CGACATTT GTTCATTTTTGAGAACTCG-3′	pYes-TAP-RLuc	Figures 3
BBV-seq -11/-20	5′**GGATCC**CAGAAAAGCG **ATG**AA GAGAAGATGGAAAAAGAATTTC-3′	5′**TCTAGA TCA**CGACATTT GTTCATTTTTGAGAACTCG-3′	pYes-TAP-RLuc	Figures 3
BBV-seq -1/-9	5′**GGATCC**ACCTAAACA **ATG**AA GAGAAGATGGAAAAAGAATTTC-3′	5′**TCTAGA TCA**CGACATTT GTTCATTTTTGAGAACTCG-3′	pYes-TAP-RLuc	Figures 3

BBV-seq Δ-29/-39	5′**GGATCC** CAAATAAAACAGAAAA GCGAACCTAAACA**ATG**AAGAGA AGATGGAAAAAGAATTTC-3′	5′**TCTAGA TCA**CGACATTT GTTCATTTTTGAGAACTCG-3′	pYes-TAP-RLuc	Figures 3
BBV-seq Δ-17/-30	5′**GGATCC**GTTTTCGAAAAAAGC GAACCTAAACA **ATG**AAGAGAA GATGGAAAAAGAATTTC-3′	5′**TCTAGA TCA**CGACATTT GTTCATTTTTGAGAACTCG-3′	pYes-TAP-RLuc	Figures 3
BBV-seq Δ-11/-20	5′**GGATCC**GTTTTCGAAACAAAT AAAACACCTAAACA **ATG**AAGAGA AGATGGAAAAAGAATTTC-3′	5′**TCTAGA TCA**CGACATTT GTTCATTTTTGAGAACTCG-3′	pYes-TAP-RLuc	Figures 3
BBV-seq Δ-1/-10	5′**GGATCC**GTTTTCGAAACAAA TAAAACAGAAAAGCGA **ATG**AAGA GAAGATGGAAAAAGAATTTC-3′	5′**TCTAGA TCA**CGACATTT GTTCATTTTTGAGAACTCG-3′	pYes-TAP-RLuc	Figures 3
TMV	5′**GGATCC**GGCCTAAGCTTGTC GACC **ATG**AAGAGAAGATGGAAA AAGAATTTC-3′	5′**TCTAGA TCA**CGACATTT GTTCATTTTTGAGAACTCG-3′	pYes-TAP-RLuc	Figure S6A
HS-BBV-seq	5′**AAGCTT**GATCCTAGGATCCTA GGATCCTAGGATCCTAGGATCCCC CCCCCCCCCCCCGTTTTCGAAACA AATAAAACAGAAAAGC-3′	5′**TCTAGA TCA**CGACATTT GTTCATTTTTGAGAACTCG-3′	pYes-H-BBV-seq-TAP-RLuc	Figure 4
pYes-TAP	5′**AAGCTT ATG**AAGAGAAGATG GAAAAAGAATTTCC-3′	5′**GGATCC TCA**CTGATGATT CGCGTCTACTTTCG3′	pBS1761	
pYes-BBV-seq-RLuc	5′**GGATCC**GTTTTCGAAACAAAT AAAACAGAAAAGCGAACCTAA ACA **ATG**ACTTCGAAAGTTTATGAT CCAGAA-3′	5′**TCTAGA TCA**CGACATTT GTTCATTTTTGAGAACTCG-3′	pJD375	
TAP-HS-BBV-seq RLuc	**5′GGATCC**GATCCTAGGATCCTA GGATCCTAGGATCCTAGGATCC CCCCCCCCCCCCCC GTTTTCGAA ACAAATAAAACAGAAAAGC-3′	5′**TCTAGA TCA**CGACATTT GTTCATTTTTGAGAACTCG-3′	pYes-BBV-seq-RLuc	Figure 5
TAP-HS-RLuc	**5′GGATCC**GATCCTAGGATCCTA GGATCCTAGGATCCTAGGATCC CCCCCCCCCCCCCC **ATG**ACTTCG AAAGTTTATGAT CCAGAA-3′	5′**TCTAGA TCA**CGACATTT GTTCATTTTTGAGAACTCG-3′	pJD375	Figure 5

Supplementary Table S3. BBV-seq and its derivatives are labeled in black. The restriction sites are bolded; the nucleotides substitutions are marked in red, sequences within coding gene regions are in blue, start and stop codons are in green and bolded. HS, hairpin-spacer.

Supplementary Figure S1

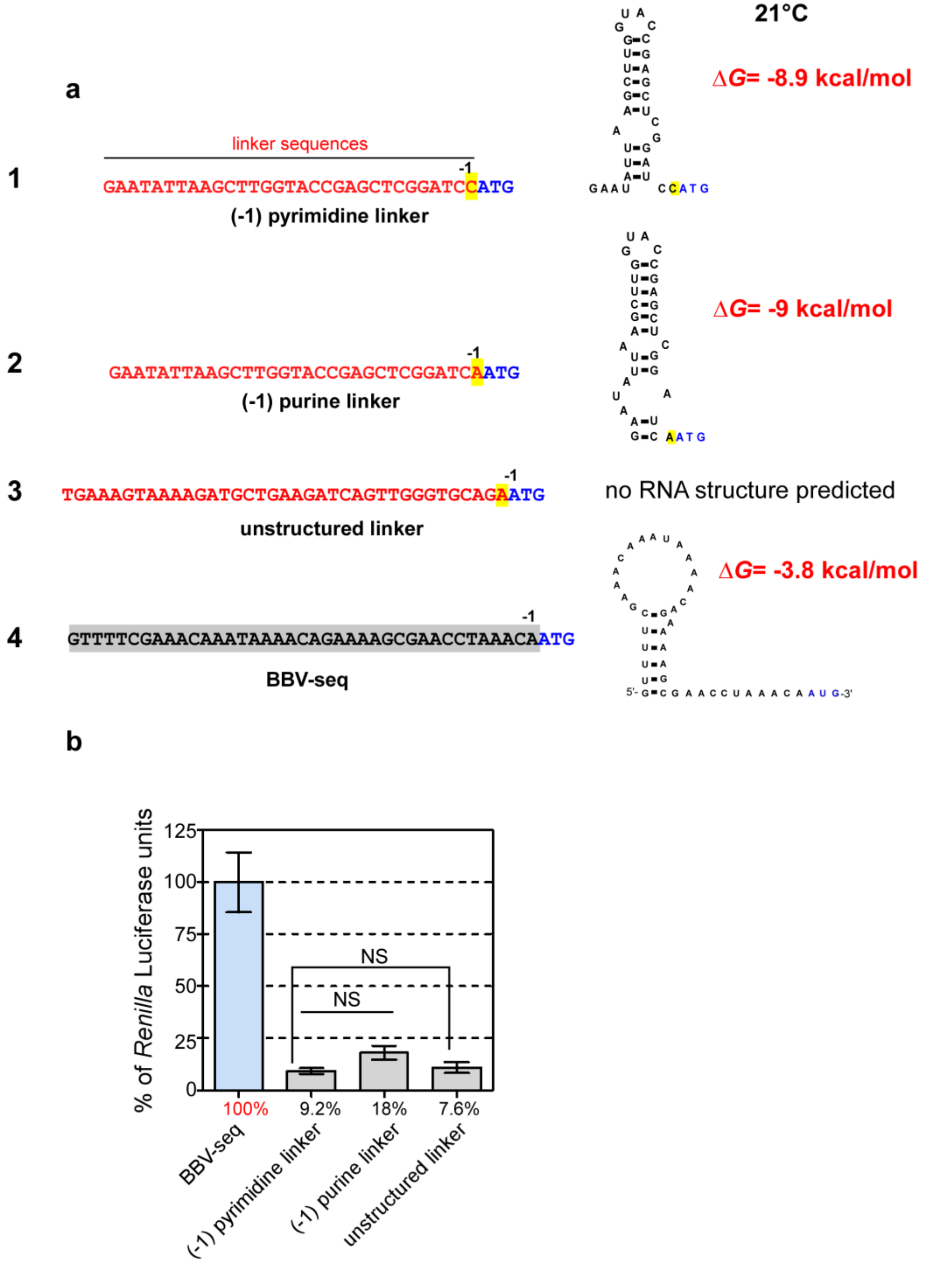

Supplementary Figure S1 (related to Figure 1)**. Neither structure nor Kozak context of the nucleotide sequence 5' to the AUG of *TAP-RLuc* are sufficient to drive reporter RNA translation. (a).** Sequences (left) and putative secondary structures determined at 21°C (right) of

the linker sequences containing a pyrimidine at position (-1) relative to AUG (1), purine at position (-1) (2), unstructured linker (3) and the control BBV-seq-5'-UTR (4). **(b).** mRNAs containing *TAP-RLuc* placed under the control of sequences described in (A) were used in cell-free yeast translation reactions, reaction products were analyzed by a *Renilla* luciferase assay. Data are presented as % *Renilla* luciferase units relative to the reaction containing *BBV-seq-TAP-RLuc* mRNA. Error bars represent standard error of the mean (SEM) of three individual experiments. Two-Tailed two-sample unequal variance *t*-test was used for statistical analysis. NS, not significant.

Supplementary Figure S2

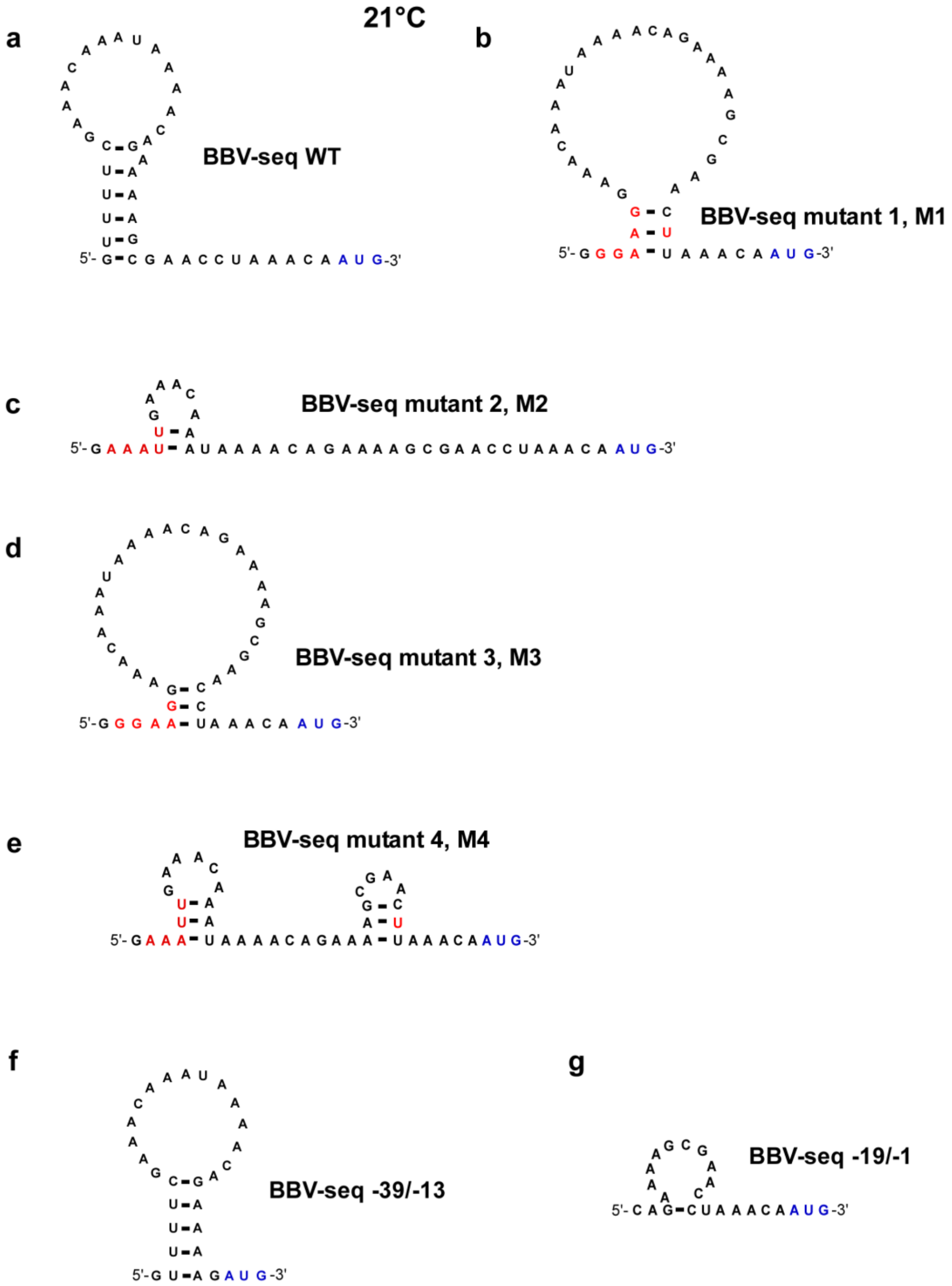

Supplementary Figure S2 (related to Figure 2). **Putative secondary structure of the BBV-seq-5'-UTR and its derivatives at 21°C.** The secondary structure of the BBV 5'-UTR WT (a) and its derivatives: mutant 1 (b), mutant 2 (c), mutant 3 (d), mutant 4 (e), -19/-1 (f) and -39/-13 (g) was predicted by the RNA structure tools version 6.0.1

(*https://rna.urmc.rochester.edu/RNAstructureWeb/*). Nucleotides substitutions in b-e are shown in red; they were designed to either enlarge (mutants 1 and 3, b&d) or reduce (mutant 2, c) the stem-loop seen at the 5'-end of the wild-type BBV-seq WT. The mutant 4 (e) is designed to place two small stem-loops at the 5'- and 3′-ends of the BBV-seq-derived sequence. Predicted secondary structure of the deletion mutants of either the 5'-end (BBV-seq -19/-39) or the 3'-end (BBV-seq -39/-13) are in (f) and (g), respectively. Start codon AUG is marked in blue.

Supplementary Figure S3

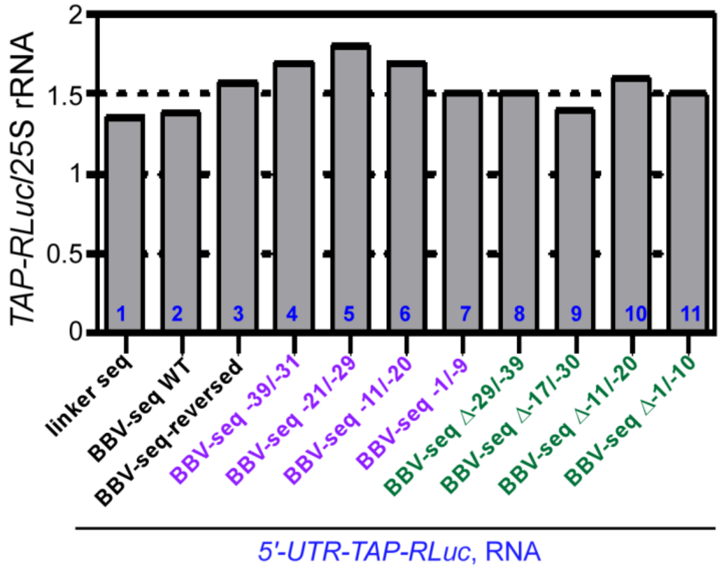

Supplementary Figure S3 (related to Figure 3). **Quantification of *TAP-RLuc* RNA carrying truncated BBV-seq 5′-UTRs shows comparable levels of mRNAs post-translation.** The hybridization signals shown on Figure 3d corresponding to the *TAP-RLuc* RNA (top) and 25S rRNA (bottom) were converted to phosphorimaging units. *TAP-RLuc* RNA was normalized to the 25S rRNA signal detected in the same sample, and the TAP-RLuc/25S rRNA ratios are presented as bar graphs.

Supplementary Figure S4

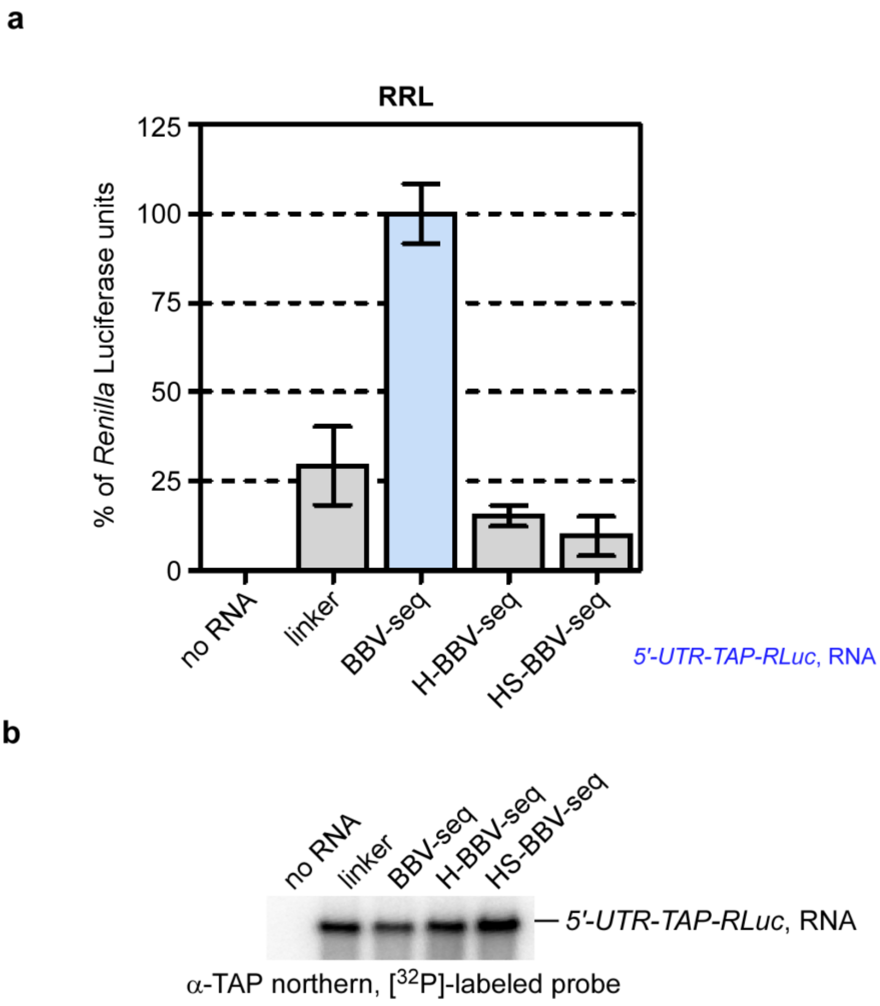

Supplementary Figure S4 (related to Figure 4). **BBV-seq-mediated translation requires a free 5′-end of RNA for translation in Rabbit Reticulocyte Lysate (RRL). (a).** RRL was charged with the indicated mRNAs and translation of the reporter TAP-RLuc was assessed by a *Renilla* Luciferase assay. Data are presented as % relative to *Renilla* Luciferase units in the reaction containing *BBV-seq- TAP-RLuc* mRNA. Error bars represent standard error of the mean (SEM) of three individual experiments. **(b).** RNA was extracted from the luciferase reactions from (a) and analyzed by northern hybridizations using *TAP*-specific probe.

Supplementary Figure S5

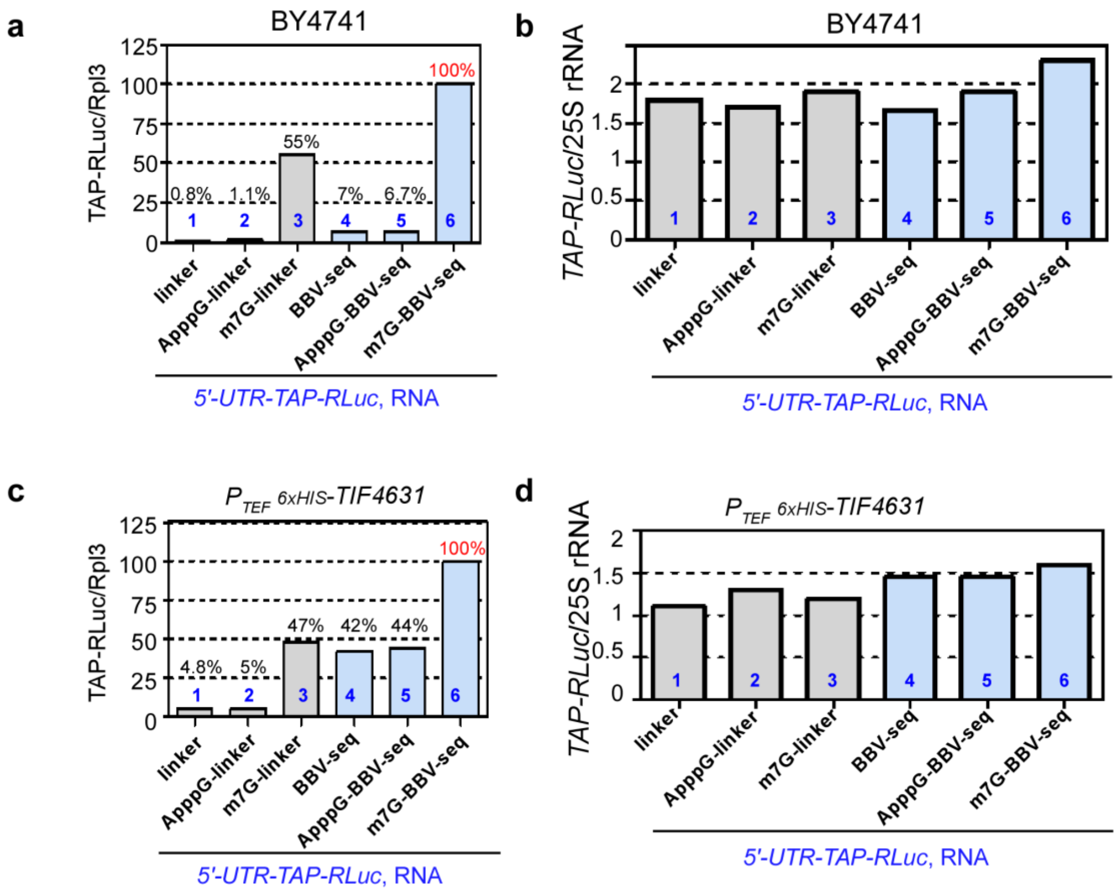

Supplementary Figure S5 (related to Figure 7a-b). **(a&c).** The fluorescent signal in the TAP-RLuc and Rpl3 bands detected by quantitative NIR western blotting shown in Figure 7a&b (top) was used to calculate TAP-RLuc normalized to Rpl3 present in the same reaction. TAP-RLuc/Rpl3 ratios are presented in the graph. TAP-RLuc/Rpl3 ratio corresponding to the reaction charged with m7G-capped *BBV-seq-TAP-RLuc* RNA is set as 100% (shown in red) and used to calculate percentage of the reporter protein in other samples (shown in black). Translationally-active lysates were prepared from wild-type strain BY4741 or from *P$_{TEF}$6xHIS-TIF4631* that expresses extra-copies of eIF4G1. **(b&d).** Northern hybridization signal corresponding to *TAP-RLuc* RNA was normalized by a signal derived from 25S rRNA present is the same sample (shown in Figure 7a&b, bottom), *TAP-RLuc*/25S rRNA ratios are presented as a bar graph.

Supplementary Figure S6

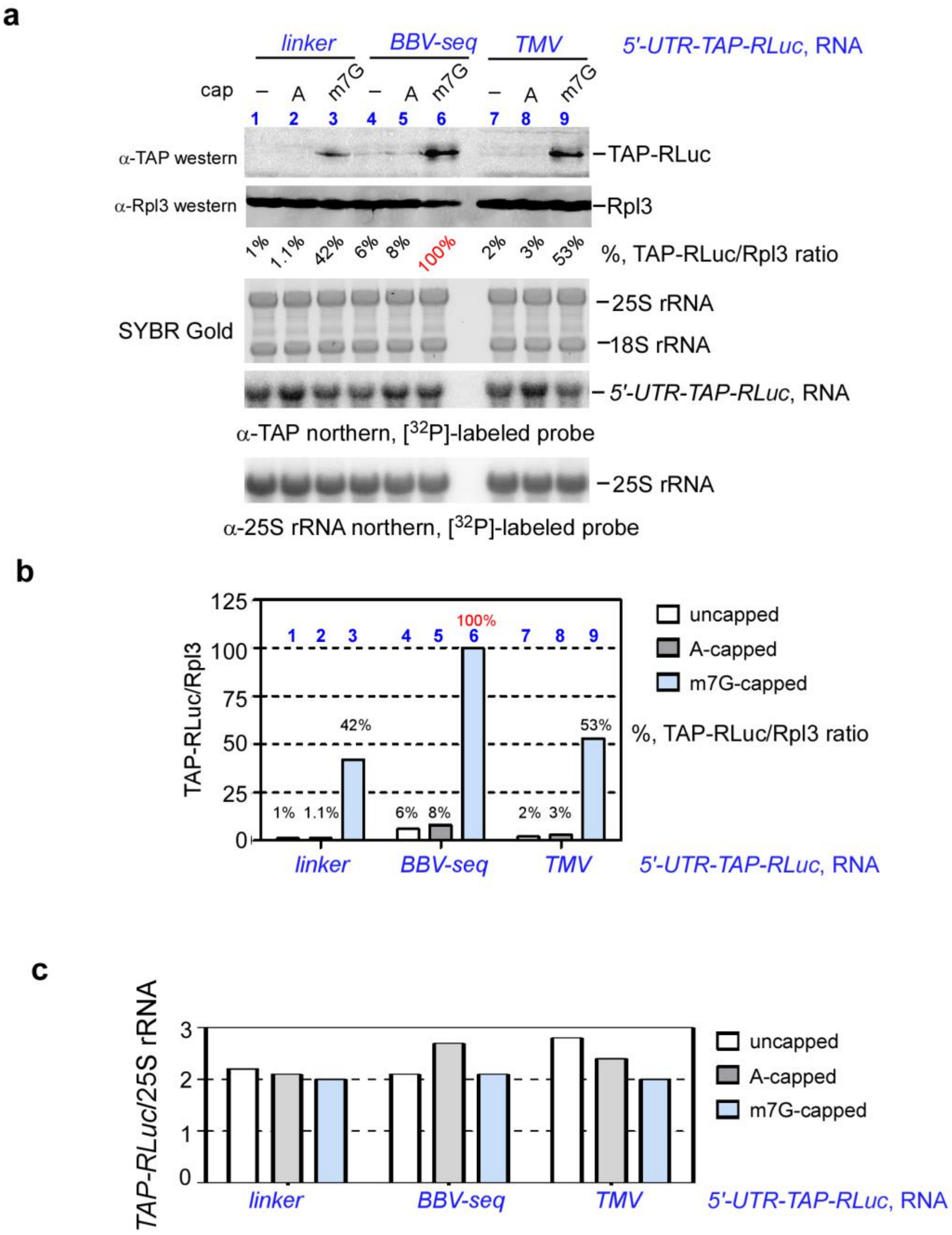

Supplementary Figure S6 (related to Figure 7a). **Cap-dependent *TMV* 5′-UTR does not initiate protein translation in cell-free reactions. (a).** mRNAs containing indicated 5′UTR were capped *in vitro* with m7G-cap (m7G), ApppG-cap (A), or remained uncapped (-) and translated in cell-free yeast lysates prepared from wild-type BY4741 strain. Translation reaction products were analyzed by quantitative NIR western blotting (top). RNA was extracted from the same

translation reactions and analyzed by northern hybridizations with the indicated probes (bottom). **(b).** The fluorescent signal in the TAP-RLuc and Rpl3 bands from (a, top) was used to calculate TAP-RLuc normalized to Rpl3 present in the same reaction, TAP-RLuc/Rpl3 ratios are presented in the graph. TAP-RLuc/Rpl3 corresponding to the reaction charged with m7G-capped *BBV-seq-TAP-RLuc* RNA is set as 100% (shown in red) and used to calculate percentage of the reporter protein in other samples (shown in black). **(c)**. Northern hybridization signal corresponding to *TAP-RLuc* RNA was normalized by a signal derived from 25S rRNA present is the same sample (shown a, bottom), *TAP-RLuc*/25S rRNA ratios are presented as a bar graph.

Supplementary Figure S7

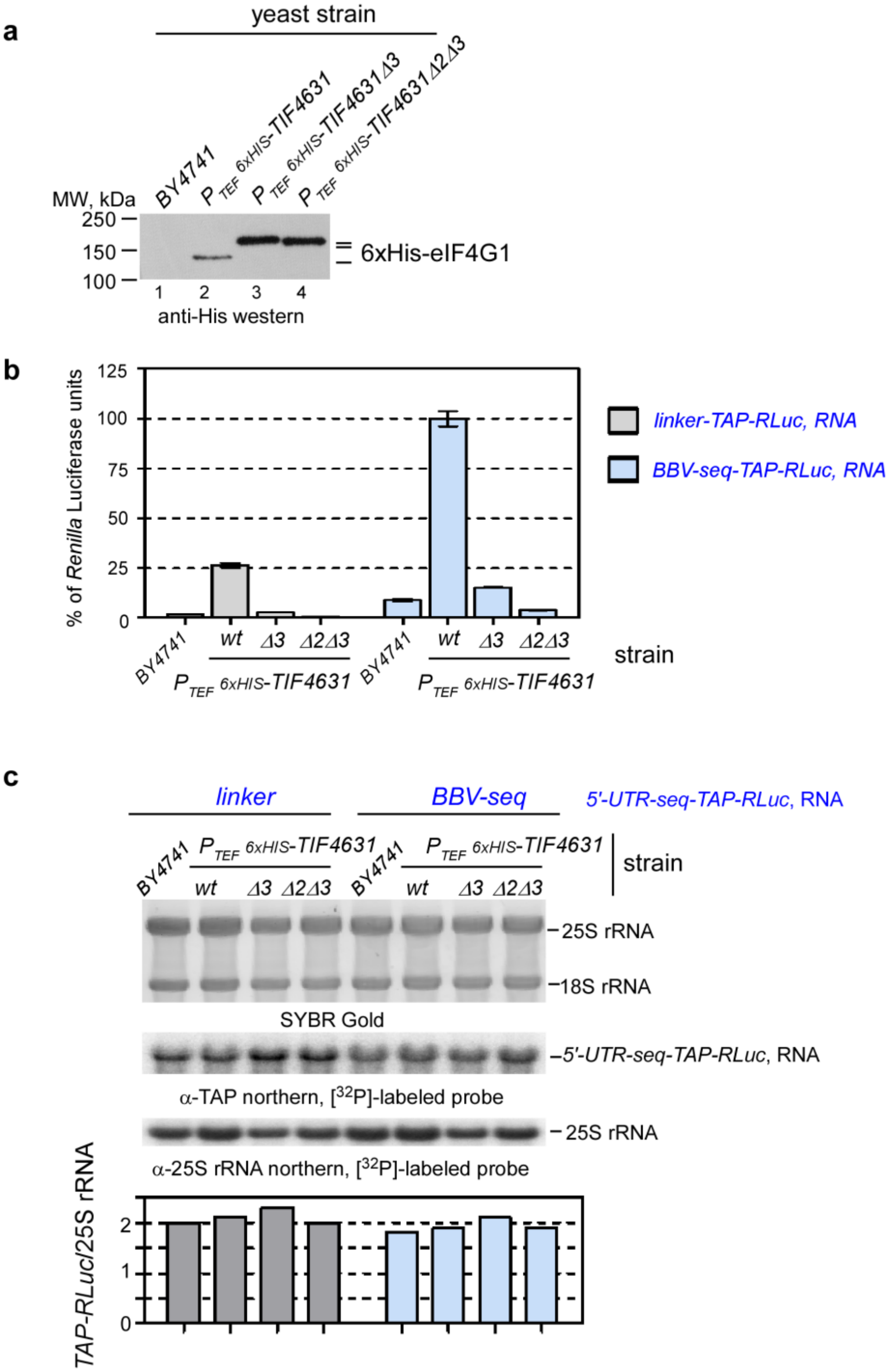

Supplementary Figure S7 (related to Figure 7c-e). **(a).** Yeast lysates prepared from wild-type (BY4741), *P$_{TEF}$-6×HIS-TIF4631* and strains containing RNA-binding domain 3 or 2/3 deletions were resolved on 8% SDS-polyacrylamide gel and subjected to conventional western blotting with anti-His antibodies. **(b).** Translationally competent yeast lysates prepared from

wild-type BY4741, *P_{TEF}*-6×*HIS-TIF4631*, *P_{TEF}*-6×*HIS-TIF4631Δ3* and *P_{TEF}*-6×*HIS-TIF4631Δ2Δ3* yeast strains were programmed with either *BBV-seq-WT-TAP-RLuc* or linker control mRNA (indicated in the figure). Protein translation was assessed by *Renilla* luciferase assay. The data, presented as bar graphs, represent % *Renilla* Luciferase units derived from the reactions performed with lysate from *P_{TEF}*-6×*HIS-TIF4631* cells programmed with *BBV-seq-WT-TAP-RLuc* mRNA. Error bars represent standard error of the mean (SEM) of three individual experiments. **(c).** RNA was extracted from the luciferase reaction from (b) and visualized in gel by SYBR Gold staining, followed by northern hybridizations with the indicated probes. The hybridization signals corresponding to the *TAP-RLuc* RNA (top) were normalized by the 25S rRNA signal (bottom) detected in the same sample and presented as the TAP-RLuc/25S rRNA ratios.

THE SOURCE FILES

Figure 1c

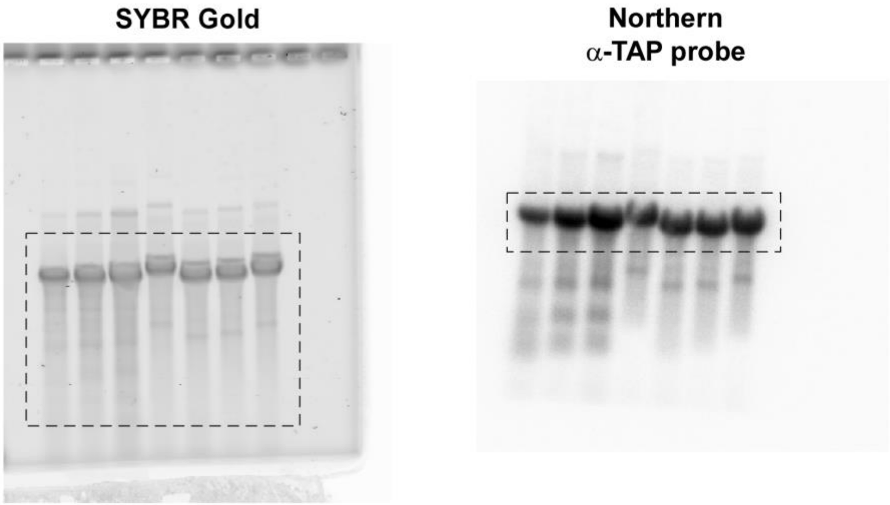

Figure 1d

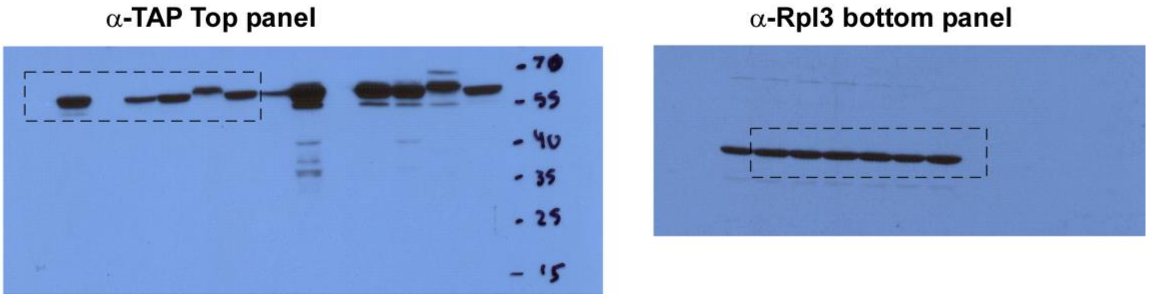

Trainor et al., Figure 1c and 1d, original data

Figure 3c

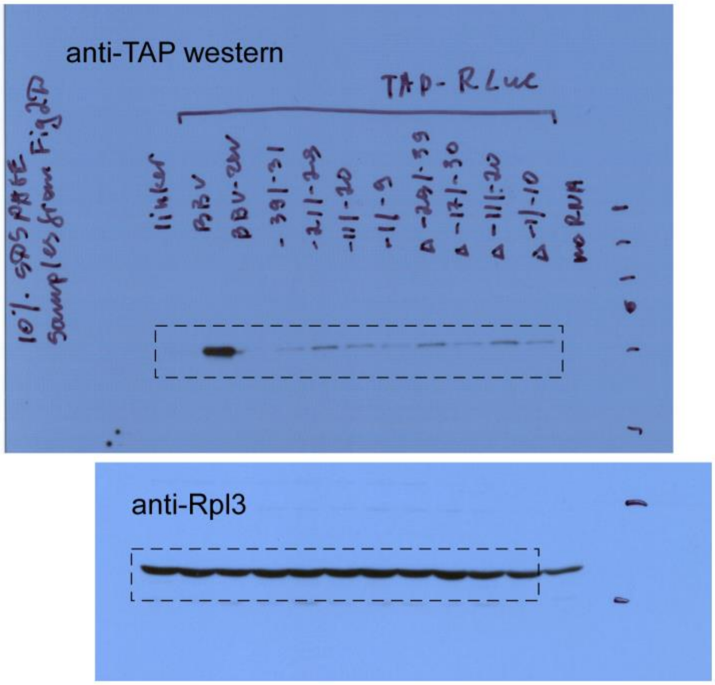

Figure 3d

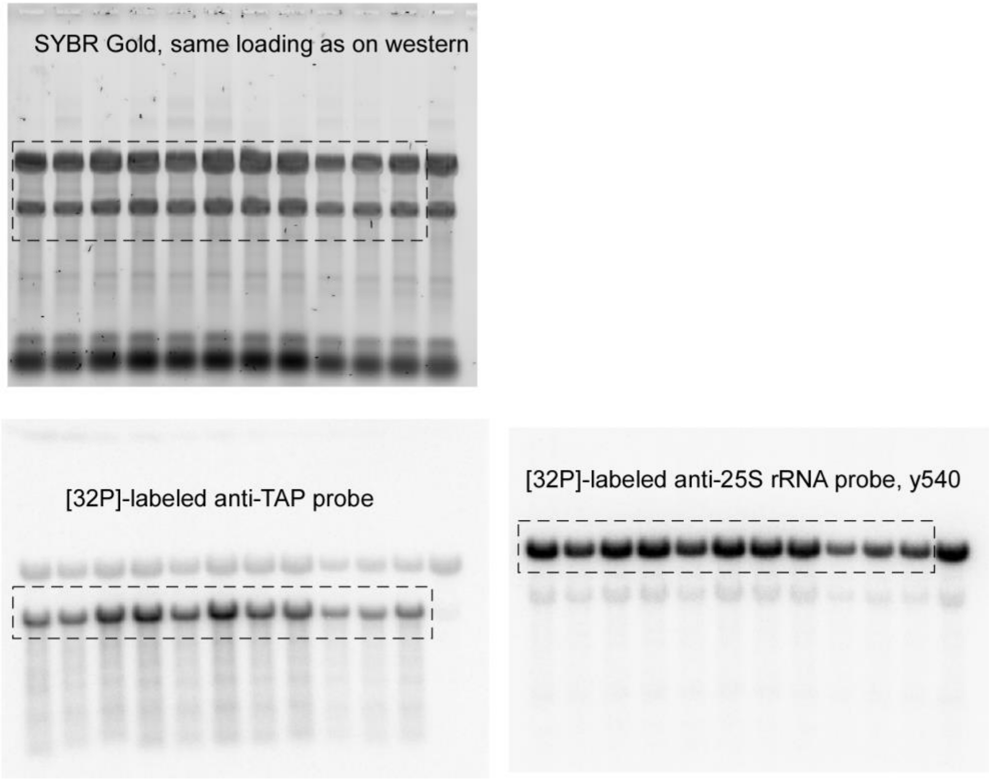

Trainor et al., Figure 3c and 3d, original data

Figure 4d

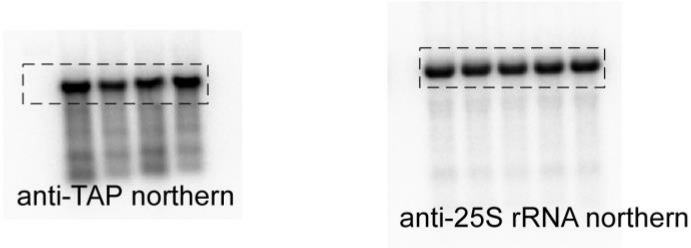

Figure 5d

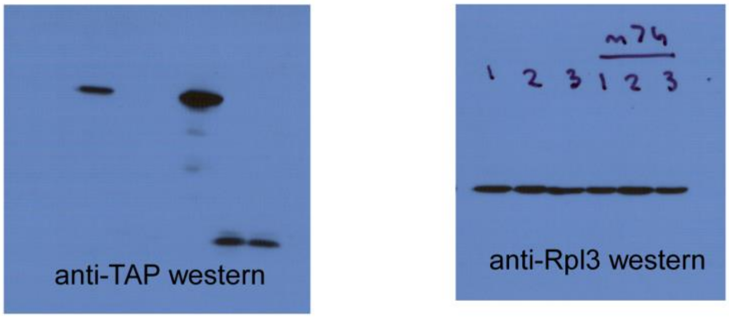

Figure 5e

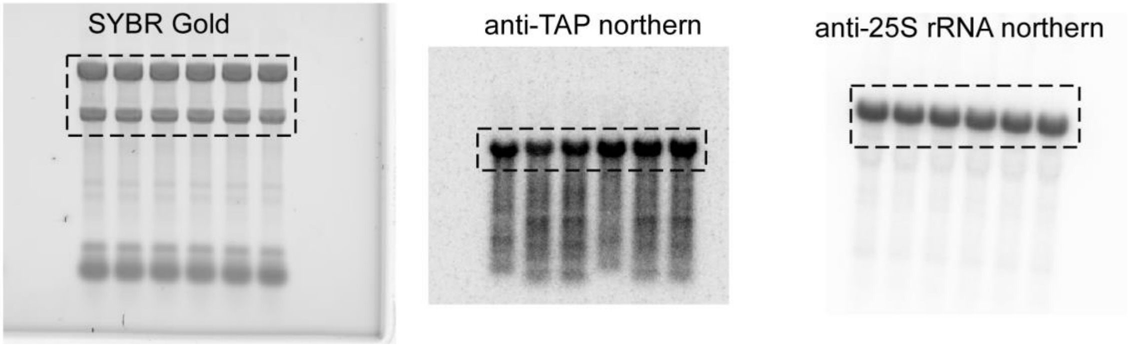

Trainor et al., Figures 4d, 5d and 5e, original data

Figure 6a

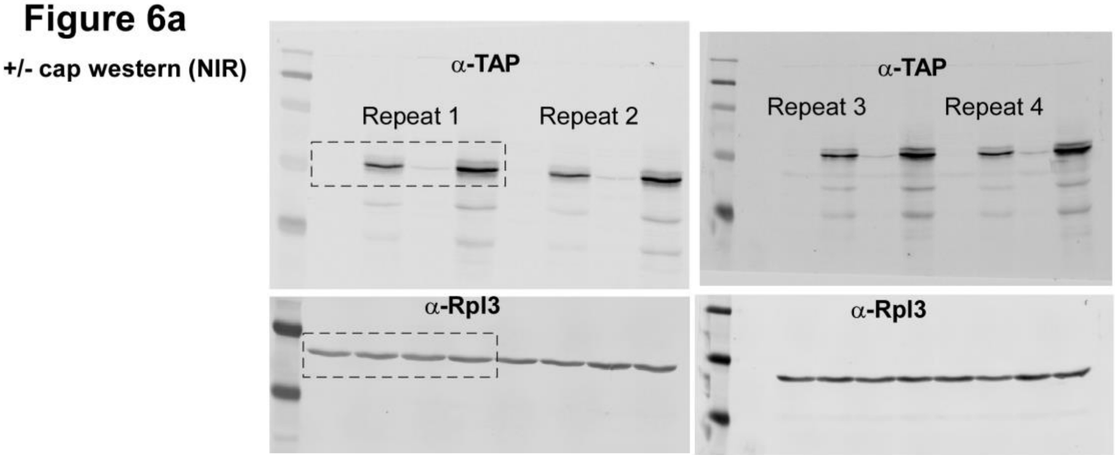

Figure 6e

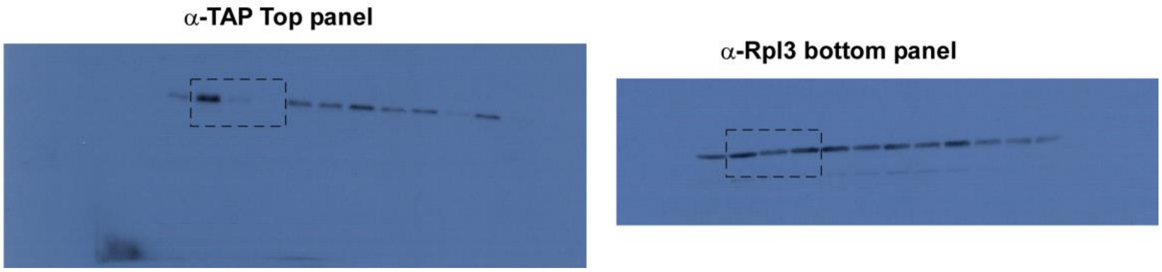

Trainor et al., Figures 6a and 6e, original data

Figure 7a

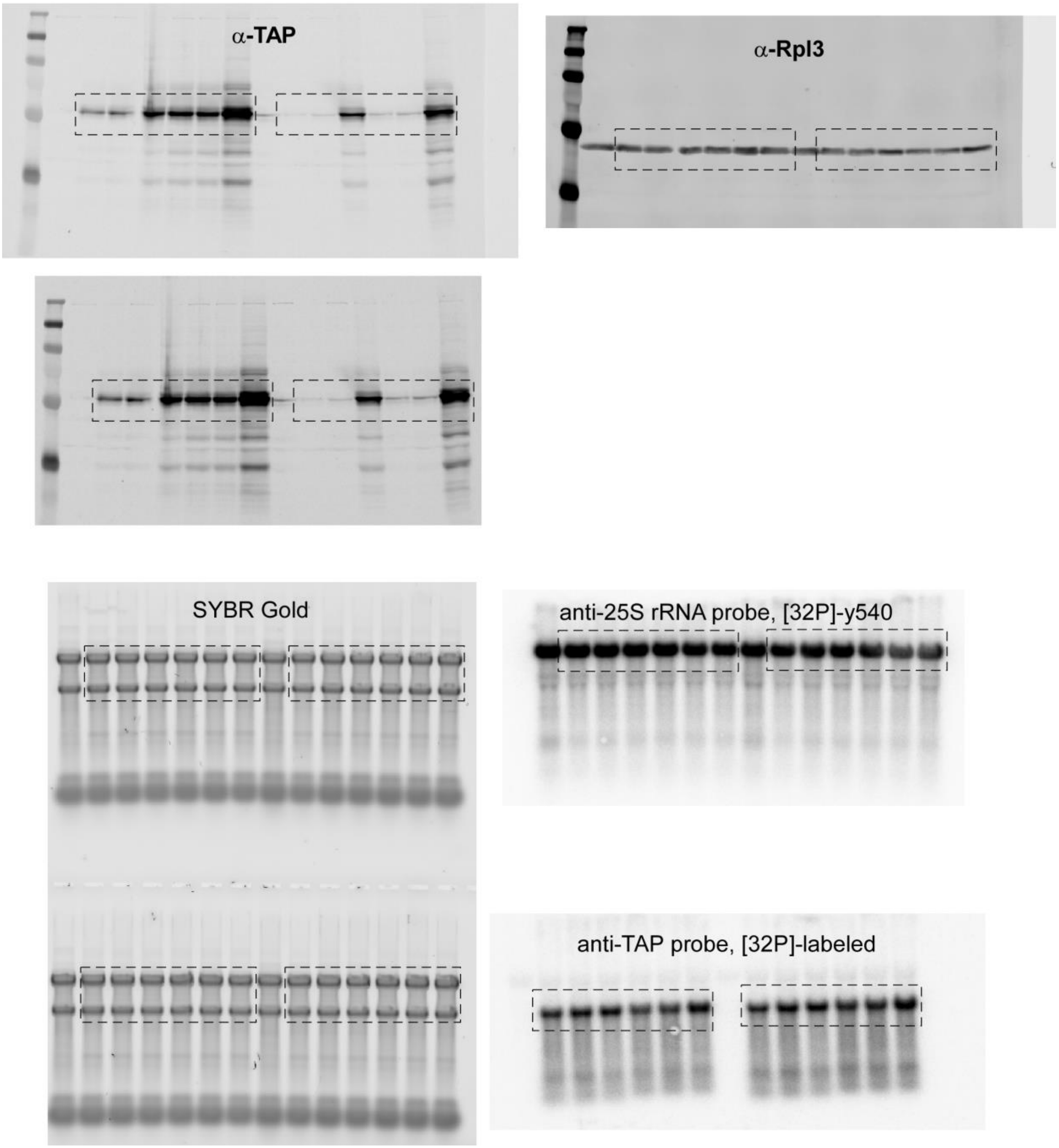

Trainor et al., Figure 7a, original data

Figure 7e and S7c

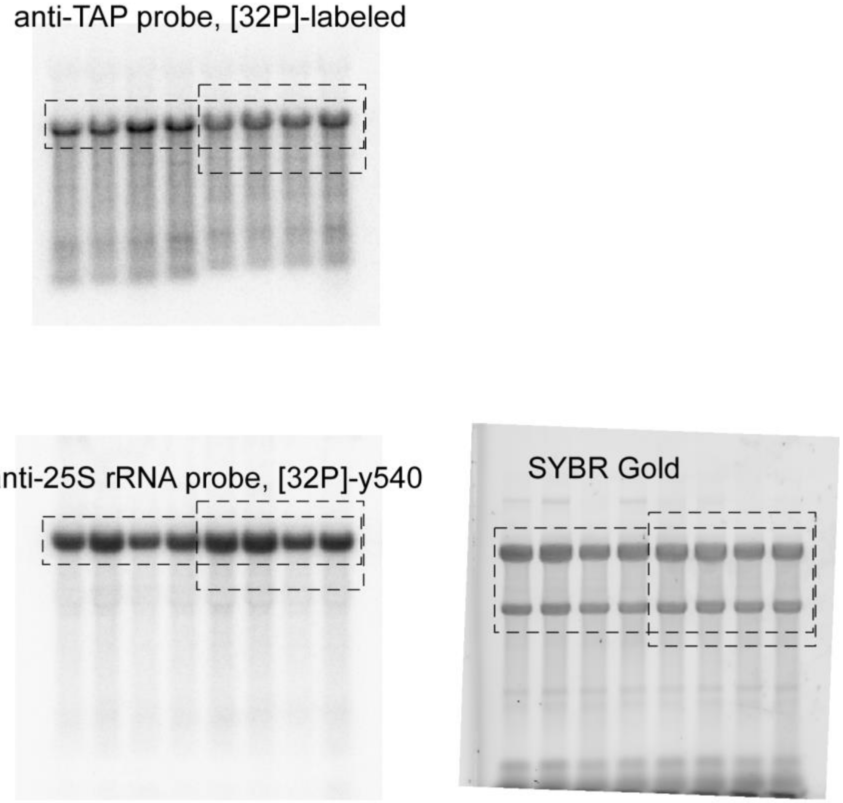

Figure S4b

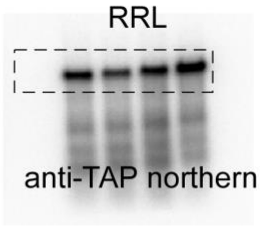

Trainor et al., Figures 7e, Supplementary Figures S7c and S4b, original data

Figure S6

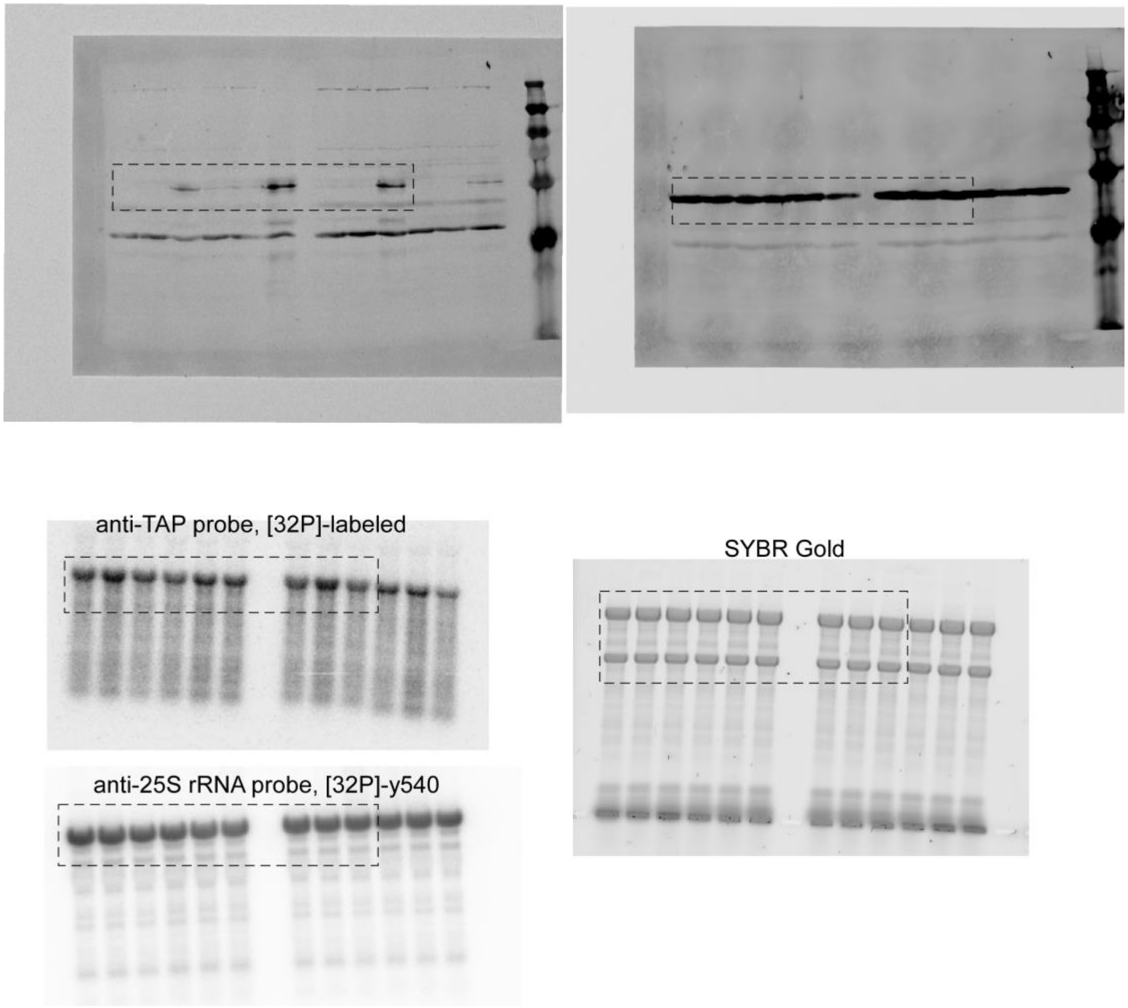

Trainor et al., Supplementary Figure S6, original data

Figure S7a

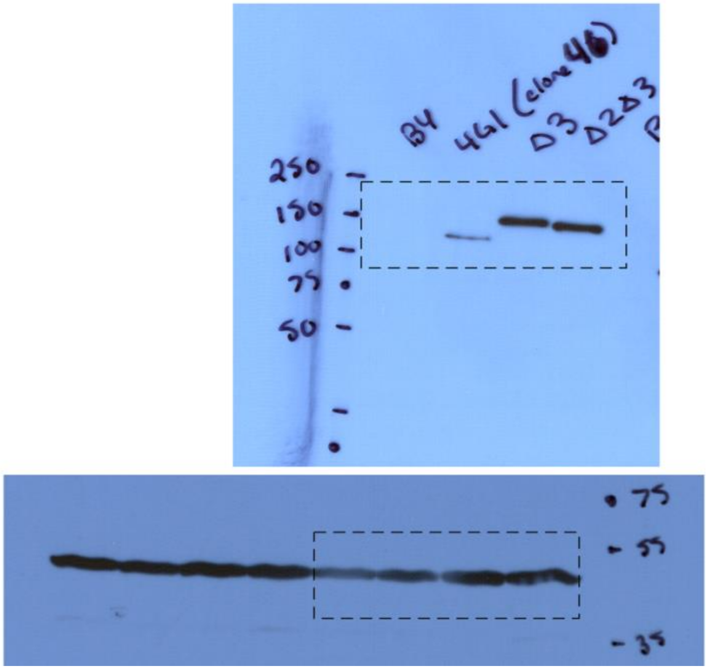

Trainor et al., Supplementary Figure S7a, original data
